# Supplementary material for: Successful ingredients of effective Collaborative Care programs in low- and middle-income countries: A rapid review
Source: Glob Ment Health (Camb). 2023 Mar 17;10:e11. doi: 10.1017/gmh.2022.60 (PMC10579696; doi:10.1017/gmh.2022.60)
Supplement: Supplementary file 1 [file S2054425122000607sup001.docx]

**Supplement 1**

**PubMed**

|  | **Concept: Integrated Care** |
| --- | --- |
| Subject Headings  (MeSH) | Delivery of Health Care, Integrated[Mesh] |
| Free text terms  (searched in [text words](https://pubmed.ncbi.nlm.nih.gov/help/#tw) [tw], journal title [ta], or subheadings [sh]) | integrated care[ta]  integrated care[tw]  shared care[tw]  collaborative care[tw]  collaborative manag*[tw]  (  (Integrat*[tw] OR coordinat*[tw] OR collaborat*[tw] OR co-locat*[tw] OR comanag*[tw] OR co-manag*[tw])  AND  care[tw]  AND  (health[tw] OR healthcare[tw])  AND  (og[sh] OR services[tw] OR delivery[tw] OR manag*[tw] OR systems[tw] OR model*[tw] OR organisational[tw] OR organizational[tw] OR quality[tw])  )  OR  ((Disease management[tw] OR Case management[tw]) AND (care[tw] OR health[tw] OR healthcare[tw]) AND (og[sh] OR services[tw] OR delivery[tw] OR model*[tw] OR quality[tw])) |

|  | **Concept: Mental Conditions** |
| --- | --- |
| Subject Headings  (MeSH) | "Mental Disorders"[Mesh]  "Mood Disorders"[Mesh]  "Anxiety Disorders"[Mesh]  "Bipolar and Related Disorders"[Mesh]  "dissociative disorders"[Mesh]  "Schizophrenia Spectrum and Other Psychotic Disorders"[Mesh]  "Trauma and Stressor Related Disorders"[Mesh]  "Substance-Related Disorders"[Mesh]  "Cocaine Smoking"[Mesh]  Methadone[Mesh]  Hallucinations[Mesh]  Mania[Mesh]  "Suicide, Attempted"[Mesh]  "Suicidal Ideation"[Mesh]  "Epilepsy"[Mesh] |
| Free text terms  (searched in [text words](https://pubmed.ncbi.nlm.nih.gov/help/#tw) [tw]) | depress*[tw]  anxiet*[tw]  "serious mental disorder*"[tw]  "serious mental illness*"[tw]  "serious mental condition*"[tw]  "serious mental disease*"[tw]  "severe mental disorder*"[tw]  "severe mental illness*"[tw]  "severe mental condition*"[tw]  "severe mental disease*"[tw]  "major mental disorder*"[tw]  "major mental illness*"[tw]  "major mental condition*"[tw]  "major mental disease*"[tw]  "drug abuse"[tw]  "drug depend*"[tw]  "substance abuse"[tw]  "substance-related disorder*"[tw]  "substance use disorder*"[tw]  "opioid use disorder*"[tw]  oud[tw]  ((cocaine OR heroin OR morphine*) AND (abuse[tw] OR depend*[tw] OR withdrawal[tw]))  methadone[tw]  addict*[tw]  psychoactive*[tw]  "drug withdrawal"[tw]  "withdrawal syndrome"[tw]  "alcoholic patient*"[tw]  "alcoholic subject*"[tw]  alcoholism[tw]  "alcohol depend*"[tw]  "alcohol-related disorder*"[tw]  "chronic ethanol*"[tw]  "chronic alcohol*"[tw]  "alcohol withdrawal"[tw]  "ethanol withdrawal"[tw]  schizophren*[tw]  schizotyp*[tw]  schizoaffective[tw]  ((delusional[tw] OR paranoid[tw]) AND disorder*[tw])  hallucination*[tw]  psychotic[tw]  schizoaffective[tw]  psychosis[tw]  psychoses[tw]  ((manic[tw] OR bipolar[tw] OR mood[tw]) AND disorder*[tw])  mania*[tw]  hypomania*[tw]  "suicide attempt*"[tw]  suicidal*[tw]  "post-traumatic stress"[tw]  "posttraumatic stress"[tw]  "stress disorder*"[tw]  epileps*[tw]  "seizure disorder*"[tw]  aura[tw]  auras[tw] |

|  | **Concept: Intervention Study** |
| --- | --- |
| Subject Headings  (MeSH) | “Clinical Trial”[pt]  “Comparative Study”[pt]  “Evaluation study”[pt]  “Cross-Over Studies”[Mesh]  “Clinical Trials as Topic”[Mesh] |
| Free text terms  (searched in [text words](https://pubmed.ncbi.nlm.nih.gov/help/#tw) [tiab]) | random*  controll*  “intervention study”  “experimental study”  “comparative study”  trial  trials  evaluat*  repeat*  compar*  versus  “before and after”  “interrupted time series” |
|  | NOT (“Animals”[Mesh] NOT (Animals[Mesh] AND “Humans”[Mesh])) |

**(**

Delivery of Health Care, Integrated[Mesh] OR integrated care[ta] OR integrated care[tw] OR shared care[tw] OR collaborative care[tw] OR collaborative manag*[tw] OR ((Integrat*[tw] OR coordinat*[tw] OR collaborat*[tw] OR co-locat*[tw] OR comanag*[tw] OR co-manag*[tw])

AND

care[tw]

AND

(health[tw] OR healthcare[tw])

AND

(og[sh] OR services[tw] OR delivery[tw] OR manag*[tw] OR systems[tw] OR model*[tw] OR organisational[tw] OR organizational[tw] OR quality[tw])

)

OR

((Disease management[tw] OR Case management[tw]) AND (care[tw] OR health[tw] OR healthcare[tw]) AND (og[sh] OR services[tw] OR delivery[tw] OR model*[tw] OR quality[tw]))

**)**

**AND**

**(**"Mental Disorders"[Mesh] OR "Mood Disorders"[Mesh] OR "Anxiety Disorders"[Mesh] OR "Bipolar and Related Disorders"[Mesh] OR "dissociative disorders"[Mesh] OR "Schizophrenia Spectrum and Other Psychotic Disorders"[Mesh] OR "Trauma and Stressor Related Disorders"[Mesh] OR "Substance-Related Disorders"[Mesh] OR "Cocaine Smoking"[Mesh] OR Methadone[Mesh] OR Hallucinations[Mesh] OR Mania[Mesh] OR "Suicide, Attempted"[Mesh] OR "Suicidal Ideation"[Mesh] OR "Epilepsy"[Mesh] OR depress*[tw] OR anxiet*[tw] OR "serious mental disorder*"[tw] OR "serious mental illness*"[tw] OR "serious mental condition*"[tw] OR "serious mental disease*"[tw] OR "severe mental disorder*"[tw] OR "severe mental illness*"[tw] OR "severe mental condition*"[tw] OR "severe mental disease*"[tw] OR "major mental disorder*"[tw] OR "major mental illness*"[tw] OR "major mental condition*"[tw] OR "major mental disease*"[tw] OR "drug abuse"[tw] OR "drug depend*"[tw] OR "substance abuse"[tw] OR "substance-related disorder*"[tw] OR "substance use disorder*"[tw] OR "opioid use disorder*"[tw] OR oud[tw] OR ((cocaine or heroin or morphine*) and (abuse[tw] or depend*[tw] or withdrawal[tw])) OR methadone[tw] OR addict*[tw] OR psychoactive*[tw] OR "drug withdrawal"[tw] OR "withdrawal syndrome"[tw] OR "alcoholic patient*"[tw] OR "alcoholic subject*"[tw] OR alcoholism[tw] OR "alcohol depend*"[tw] OR "alcohol-related disorder*"[tw] OR "chronic ethanol*"[tw] OR "chronic alcohol*"[tw] OR "alcohol withdrawal"[tw] OR "ethanol withdrawal"[tw] OR schizophren*[tw] OR schizotyp*[tw] OR schizoaffective[tw] OR ((delusional[tw] or paranoid[tw]) and disorder*[tw]) OR hallucination*[tw] OR psychotic[tw] OR schizoaffective[tw] OR psychosis[tw] OR psychoses[tw] OR ((manic[tw] or bipolar[tw] or mood[tw]) and disorder*[tw]) OR mania*[tw] OR hypomania*[tw] OR "suicide attempt*"[tw] OR suicidal*[tw] OR "post-traumatic stress"[tw] OR "posttraumatic stress"[tw] OR "stress disorder*"[tw] OR epileps*[tw] OR "seizure disorder*"[tw] OR aura[tw] OR auras[tw]**)**

**AND**

**(**“Clinical Trial”[pt] OR “Comparative Study”[pt] OR “Evaluation study”[pt] OR “Cross-Over Studies”[Mesh] OR “Clinical Trials as Topic”[Mesh] OR random*[tiab] OR controll*[tiab] OR “intervention study”[tiab] OR “experimental study”[tiab] OR “comparative study”[tiab] OR trial[tiab] OR trials[tiab] OR evaluat*[tiab] OR repeat*[tiab] OR compar*[tiab] OR versus[tiab] OR “before and after”[tiab] OR “interrupted time series”[tiab]**)**

NOT (“Animals”[Mesh] NOT (Animals[Mesh] AND “Humans”[Mesh]))

NOT (child[Mesh] NOT adult[Mesh])

**AND**

**(**

Afghanistan[Mesh] OR Albania[Mesh] OR Algeria[Mesh] OR American Samoa[Mesh] OR Angola[Mesh] OR Antigua and Barbuda[Mesh] OR Argentina[Mesh] OR Armenia[Mesh] OR Aruba[Mesh] OR Azerbaijan[Mesh] OR Bahrain[Mesh] OR Bangladesh[Mesh] OR Barbados[Mesh] OR Republic of Belarus[Mesh] OR Belize[Mesh] OR Benin[Mesh] OR Bhutan[Mesh] OR Bolivia[Mesh] OR Bosnia and Herzegovina[Mesh] OR Botswana[Mesh] OR Brazil[Mesh] OR Bulgaria[Mesh] OR Burkina Faso[Mesh] OR Burundi[Mesh] OR Cabo Verde[Mesh] OR Cambodia[Mesh] OR Cameroon[Mesh] OR Central African Republic[Mesh] OR Chad[Mesh] OR Chile[Mesh] OR China[Mesh] OR Colombia[Mesh] OR Comoros[Mesh] OR Democratic Republic of The Congo[Mesh] OR Congo[Mesh] OR Costa Rica[Mesh] OR Cote D’ivoire[Mesh] OR Croatia[Mesh] OR Cuba[Mesh] OR Cyprus[Mesh] OR Czech Republic[Mesh] OR Djibouti[Mesh] OR Dominica[Mesh] OR Dominican Republic[Mesh] OR Ecuador[Mesh] OR Egypt[Mesh] OR El Salvador[Mesh] OR Equatorial Guinea[Mesh] OR Eritrea[Mesh] OR Estonia[Mesh] OR Swaziland[Mesh] OR Ethiopia[Mesh] OR Fiji[Mesh] OR Gabon[Mesh] OR Gambia[Mesh] OR Georgia (Republic)[Mesh] OR Ghana[Mesh] OR Gibraltar[Mesh] OR Greece[Mesh] OR Grenada[Mesh] OR Guam[Mesh] OR Guatemala[Mesh] OR Guinea[Mesh] OR Guinea Bissau[Mesh] OR Guyana[Mesh] OR Haiti[Mesh] OR Honduras[Mesh] OR Hungary[Mesh] OR India[Mesh] OR Indonesia[Mesh] OR Iran[Mesh] OR Iraq[Mesh] OR Jamaica[Mesh] OR Jordan[Mesh] OR Kazakhstan[Mesh] OR Kenya[Mesh] OR Democratic People’s Republic of Korea[Mesh] OR Republic of Korea[Mesh] OR Kosovo[Mesh] OR Kyrgyzstan[Mesh] OR Laos[Mesh] OR Latvia[Mesh] OR Lebanon[Mesh] OR Lesotho[Mesh] OR Liberia[Mesh] OR Libya[Mesh] OR Lithuania[Mesh] OR Macau[Mesh] OR Republic of North Macedonia[Mesh] OR Madagascar[Mesh] OR Malawi[Mesh] OR Malaysia[Mesh] OR Indian Ocean Islands[Mesh] OR Mali[Mesh] OR Malta[Mesh] OR Micronesia[Mesh] OR Palau[Mesh] OR Mauritania[Mesh] OR Mauritius[Mesh] OR Mexico[Mesh] OR Moldova[Mesh] OR Mongolia[Mesh] OR Montenegro[Mesh] OR Morocco[Mesh] OR Mozambique[Mesh] OR Myanmar[Mesh] OR Namibia[Mesh] OR Nepal[Mesh] OR Netherlands Antilles[Mesh] OR Nicaragua[Mesh] OR Niger[Mesh] OR Nigeria[Mesh] OR Oman[Mesh] OR Pakistan[Mesh] OR Panama[Mesh] OR Papua New Guinea[Mesh] OR Paraguay[Mesh] OR Peru[Mesh] OR Philippines[Mesh] OR Poland[Mesh] OR Portugal[Mesh] OR Puerto Rico[Mesh] OR Romania[Mesh] OR Russia[Mesh] OR Rwanda[Mesh] OR Samoa[Mesh] OR Sao Tome and Principe[Mesh] OR Saudi Arabia[Mesh] OR Senegal[Mesh] OR Serbia[Mesh] OR Seychelles[Mesh] OR Sierra Leone[Mesh] OR Slovakia[Mesh] OR Slovenia[Mesh] OR Melanesia[Mesh] OR Somalia[Mesh] OR South Africa[Mesh] OR South Sudan[Mesh] OR Sri Lanka[Mesh] OR Saint Kitts and Nevis[Mesh] OR Saint Lucia[Mesh] OR Saint Vincent and The Grenadines[Mesh] OR Sudan[Mesh] OR Suriname[Mesh] OR Syria[Mesh] OR Tajikistan[Mesh] OR Tanzania[Mesh] OR Thailand[Mesh] OR Timor Leste[Mesh] OR Togo[Mesh] OR Tonga[Mesh] OR Trinidad and Tobago[Mesh] OR Tunisia[Mesh] OR Turkey[Mesh] OR Turkmenistan[Mesh] OR Uganda[Mesh] OR Ukraine[Mesh] OR Uruguay[Mesh] OR Uzbekistan[Mesh] OR Vanuatu[Mesh] OR Venezuela[Mesh] OR Vietnam[Mesh] OR Middle East[Mesh] OR Yemen[Mesh] OR Yugoslavia[Mesh] OR Zambia[Mesh] OR Zimbabwe[Mesh] OR Africa South of The Sahara[Mesh] OR Africa, Central[Mesh] OR Africa, Northern[Mesh] OR Africa, Southern[Mesh] OR Africa, Eastern[Mesh] OR Africa, Western[Mesh] OR West Indies[Mesh] OR Indian Ocean Islands[Mesh] OR Caribbean Region[Mesh] OR Central America[Mesh] OR Latin America[Mesh] OR South America[Mesh] OR Asia, Central[Mesh] OR Asia, Northern[Mesh] OR Asia, Southeastern[Mesh] OR Asia, Western[Mesh] OR Europe, Eastern[Mesh] OR Developing Countries[Mesh] OR

Afghan*[tw] OR Albania*[tw] OR Algeria[tw] OR "American Samoa*"[tw] OR Angola*[tw] OR Antigua*[tw] OR Barbuda*[tw] OR Argentin*[tw] OR Armenia*[tw] OR Aruba*[tw] OR Azerbaijan*[tw] OR Bahrain*[tw] OR Bangladesh*[tw] OR Bangalees[tw] OR Bajan*[tw] OR Barbados[tw] OR Belarus*[tw] OR Byelarus*[tw] OR Belorussia*[tw] OR Byelorussia*[tw] OR Belize*[tw] OR British Honduras[tw] OR Benin*[tw] OR Dahomey[tw] OR Bhutan*[tw] OR Bolivia*[tw] OR Bosnia*[tw] OR Hercegovina*[tw] OR Herzegovina*[tw] OR Botswana*[tw] OR Batswana[tw] OR Brazil*[tw] OR Brasil*[tw] OR Bulgaria[tw] OR Burkina Faso[tw] OR Burkina Fasso[tw] OR Burkinabe[tw] OR Burkinese[tw] OR Upper Volta[tw] OR Burundi*[tw] OR Urundi[tw] OR Cabo Verde*[tw] OR Cape Verde*[tw] OR Cambodia*[tw] OR Kampuchea[tw] OR Khmer[tw] OR Cameroon*[tw] OR Cameron*[tw] OR Cameroun*[tw] OR "Central African"[tw] OR Ubangi Shari[tw] OR Chad[tw] OR Chadian*[tw] OR Chile*[tw] OR China[tw] OR Chinese[tw] OR Colombia*[tw] OR Comoros[tw] OR Comores[tw] OR Comoro Islands[tw] OR Comorian*[tw] OR Mayotte[tw] OR Congo[tw] OR Congolese[tw] OR Zaire[tw] OR Costa Rica*[tw] OR Cote D’ivoire[tw] OR Cote D’ Ivoire[tw] OR Cote Divoire[tw] OR Cote D Ivoire[tw] OR Ivory Coast[tw] OR Ivorian*[tw] OR Croatia*[tw] OR Cuba[tw] OR Cuban*[tw] OR Cyprus[tw] OR Cypriot*[tw] OR Czech*[tw] OR Djibouti*[tw] OR French Somaliland[tw] OR Dominica*[tw] OR Ecuador*[tw] OR Egypt*[tw] OR United Arab Republic[tw] OR El Salvador[tw] OR Salvadoran*[tw] OR "Equatorial Guinea*"[tw] OR Equatoguinean*[tw] OR Spanish Guinea[tw] OR Eritrea*[tw] OR Estonia*[tw] OR Eswatini[tw] OR Swaziland[tw] OR Swazi[tw] OR Swazis[tw] OR Swati[tw] OR Swatis[tw] OR Ethiopia*[tw] OR Fiji*[tw] OR Gabon*[tw] OR Gambia*[tw] OR Georgia*[tw] OR Ghana*[tw] OR Gold Coast[tw] OR Gibraltar*[tw] OR Greece[tw] OR Greek*[tw] OR Grenada*[tw] OR Guam[tw] OR Guamanian*[tw] OR Guatemala[tw] OR Guatemalan*[tw] OR Guinea*[tw] OR Guyan*[tw] OR Guian*[tw] OR Haiti*[tw] OR Hispaniola[tw] OR Hondura*[tw] OR Hungar*[tw] OR Indian[tw] OR Indians[tw] OR India[tw] OR Indonesia*[tw] OR Timor*[tw] OR Iran*[tw] OR Iraq*[tw] OR Isle Of Man[tw] OR Manx[tw] OR Jamaica*[tw] OR Jordan*[tw] OR Kazakh*[tw] OR Kenya*[tw] OR Korea*[tw] OR Kosovo*[tw] OR Kosovar*[tw] OR Kyrgyz*[tw] OR Kirghiz*[tw] OR Lao[tw] OR Laos[tw] OR Laotian*[tw] OR Latvia*[tw] OR Lebanon[tw] OR Lebanese[tw] OR Lesoth*[tw] OR Basutoland[tw] OR Mosotho[tw] OR Basotho[tw] OR Liberia*[tw] OR Libya*[tw] OR Lithuania*[tw] OR Macau[tw] OR Macao[tw] OR Macanese[tw] OR Macedonia*[tw] OR Madagasca*[tw] OR Malagasy Republic[tw] OR Malawi*[tw] OR Nyasaland[tw] OR Malay*[tw] OR Maldiv*[tw] OR Indian Ocean[tw] OR Mali[tw] OR Malian*[tw] OR Malta[tw] OR Maltese[tw] OR Micronesia*[tw] OR Kirabati*[tw] OR Marshall Islands[tw] OR Marshallese[tw] OR Nauru*[tw] OR Northern Mariana Islands[tw] OR Palau*[tw] OR Tuvalu*[tw] OR Mauritania[tw] OR Mauritius[tw] OR Mauritanian*[tw] OR Mauritian*[tw] OR Mexic*[tw] OR Moldov*[tw] OR Mongol*[tw] OR Montenegr*[tw] OR Morocc*[tw] OR Ifni[tw] OR Mozambique[tw] OR Mozambican*[tw] OR Portuguese East Africa[tw] OR Myanma*[tw] OR Burma[tw] OR Burmese[tw] OR Namibia*[tw] OR Nepal*[tw] OR Netherlands Antille*[tw] OR Nicaragua*[tw] OR Niger*[tw] OR Mariana*[tw] OR Oman[tw] OR Omani[tw] OR Muscat[tw] OR Pakistan*[tw] OR Palestin*[tw] OR Panama*[tw] OR Papua New Guinea*[tw] OR Paraguay*[tw] OR Peru[tw] OR Peruvian*[tw] OR Philipine*[tw] OR Philippine*[tw] OR Phillipine*[tw] OR Phillippine*[tw] OR Filipino*[tw] OR Filipina*[tw] OR Poland[tw] OR Polish[tw] OR Portug*[tw] OR "Puerto Ric*"[tw] OR Romania*[tw] OR Russia*[tw] OR Ussr[tw] OR Soviet*[tw] OR Rwand*[tw] OR Ruand*[tw] OR Samoa*[tw] OR Pacific Islands[tw] OR Polynesia[tw] OR Samoan Islands[tw] OR Sao Tome And Principe[tw] OR Sao Tomean*[tw] OR Santomean*[tw] OR Saudi*[tw] OR Senegal*[tw] OR Serbia*[tw] OR Seychell*[tw] OR "Sierra Leone*"[tw] OR Slovak*[tw] OR Slovenia*[tw] OR Melanesia*[tw] OR "Solomon Island*"[tw] OR "Norfolk Island*"[tw] OR Somali*[tw] OR "South Africa*"[tw] OR "South Sudan*"[tw] OR "Sri Lanka*"[tw] OR Ceylon*[tw] OR Saint Kitts And Nevis[tw] OR St Kitts And Nevis[tw] OR Kittitian*[tw] OR Nevisian*[tw] OR "Saint Lucia*"[tw] OR St Lucia[tw] OR Saint Vincent[tw] OR St Vincent[tw] OR Vincentian*[tw] OR Grenadines[tw] OR Sudan[tw] OR Sudanese[tw] OR Surinam*[tw] OR Syria[tw] OR Syrian*[tw] OR Tajik*[tw] OR Tadjik*[tw] OR Tadzhik*[tw] OR Tanzania*[tw] OR Tanganyika*[tw] OR Thai[tw] OR Thailand[tw] OR Siam[tw] OR Timor Leste[tw] OR East Timor[tw] OR Timorese*[tw] OR Togo[tw] OR Togolese[tw] OR Tonga[tw] OR Tongan*[tw] OR Trinidad*[tw] OR Tobago*[tw] OR Tunisia*[tw] OR Turkey[tw] OR Turkmen*[tw] OR Uganda*[tw] OR Ukrain*[tw] OR Uruguay*[tw] OR Uzbek*[tw] OR Vanuatu*[tw] OR "New Hebride*"[tw] OR Venezuela*[tw] OR Vietnam*[tw] OR Viet Nam[tw] OR Middle East[tw] OR West Bank[tw] OR Gaza[tw] OR Yemen*[tw] OR Yugoslav*[tw] OR Zambia*[tw] OR Zimbabwe*[tw] OR "Northern Rhodesia*"[tw] OR

global south[tw] OR Africa south of the sahara[tw] OR Saharan Africa[tw] OR subSaharan Africa[tw] OR central Africa[tw] OR north Africa[tw] OR northern Africa[tw] OR Magreb[tw] OR Maghreb[tw] OR Sahara[tw] OR southern Africa[tw] OR east Africa[tw] OR eastern Africa[tw] OR west Africa[tw] OR western Africa[tw] OR West Indies[tw] OR Indian Ocean islands[tw] OR Caribbean[tw] OR Central America[tw] OR Latin America[tw] OR South America[tw] OR Central Asia[tw] OR north Asia[tw] OR northern Asia[tw] OR southeastern Asia[tw] OR south eastern Asia[tw] OR southeast Asia[tw] OR south east Asia[tw] OR western Asia[tw] OR east Europe[tw] OR eastern Europe[tw] OR

developing country[tw] OR developing countries[tw] OR developing nation[tw] OR developing nations[tw] OR developing population[tw] OR developing populations[tw] OR developing world[tw] OR less developed country[tw] OR less developed countries[tw] OR less developed nation[tw] OR less developed nations[tw] OR less developed world[tw] OR lesser developed countries[tw] OR lesser developed nations[tw] OR under developed country[tw] OR under developed countries[tw] OR under developed nations[tw] OR under developed world[tw] OR underdeveloped country[tw] OR underdeveloped countries[tw] OR underdeveloped nation[tw] OR underdeveloped nations[tw] OR underdeveloped population[tw] OR underdeveloped populations[tw] OR underdeveloped world[tw] OR middle income country[tw] OR middle income countries[tw] OR middle income nation[tw] OR middle income nations[tw] OR middle income population[tw] OR middle income populations[tw] OR low income country[tw] OR low income countries[tw] OR low income nation[tw] OR low income nations[tw] OR low income population[tw] OR low income populations[tw] OR lower income country[tw] OR lower income countries[tw] OR lower income nations[tw] OR lower income population[tw] OR lower income populations[tw] OR underserved countries[tw] OR underserved nations[tw] OR underserved population[tw] OR underserved populations[tw] OR under served population[tw] OR under served populations[tw] OR deprived countries[tw] OR deprived population[tw] OR deprived populations[tw] OR poor country[tw] OR poor countries[tw] OR poor nation[tw] OR poor nations[tw] OR poor population[tw] OR poor populations[tw] OR poor world[tw] OR poorer countries[tw] OR poorer nations[tw] OR poorer population[tw] OR poorer populations[tw] OR developing economy[tw] OR developing economies[tw] OR less developed economy[tw] OR less developed economies[tw] OR underdeveloped economies[tw] OR middle income economy[tw] OR middle income economies[tw] OR low income economy[tw] OR low income economies[tw] OR lower income economies[tw] OR low gdp[tw] OR low gnp[tw] OR low gross domestic[tw] OR low gross national[tw] OR lower gdp[tw] OR lower gross domestic[tw] OR lmic[tw] OR lmics[tw] OR third world[tw] OR lami country[tw] OR lami countries[tw] OR transitional country[tw] OR transitional countries[tw] OR emerging economies[tw] OR emerging nation[tw] OR emerging nations[tw]

**)**

1,261 results as of 5/23/22

**Embase; Elsevier**

|  | **Concept: Integrated Care** |
| --- | --- |
| Subject Headings  (Emtree) | 'integrated health care system'/exp  'collaborative care team'/exp |
| Free text terms  (searched in journal title (:jt) | 'integrated care':jt |
| Free text terms  (searched in title, abstract, and keyword (:ti,ab,kw)) | 'integrated care'  'shared care'  'collaborative care'  'collaborative manag*'  ((Integrat* OR coordinat* OR collaborat* OR co-locat* OR comanag* OR co-manag*) AND care AND (health OR healthcare) AND (services OR delivery OR manag* OR systems OR model* OR organisational OR organizational OR quality))  (('disease management' OR 'case management') AND (care OR health OR healthcare) AND (services OR delivery OR model* OR quality)) |

|  | **Concept: Mental Conditions** |
| --- | --- |
| Subject Headings  (Emtree) | 'mental disease'/exp  'mood disorder'/exp  'anxiety disorder'/exp  'dissociative disorder'/exp  'psychosis'/exp  'drug dependence'/exp  'cocaine smoking'/exp  'methadone'/exp  'withdrawal syndrome'/exp  'mania'/exp  'suicide attempt'/exp  'suicidal ideation'/exp  'epilepsy'/exp |
| Free text terms  (searched in title, abstract, and keyword (:ti,ab,kw)) | depress*  anxiet*  ((serious OR severe OR major) NEXT/1 mental NEXT/1 (disorder* OR illness* OR condition* OR disease*))  'drug abuse'  'drug depend*'  'substance abuse'  'substance-related disorder*'  'substance use disorder*'  'opioid use disorder*'  oud  ((cocaine OR heroin OR morphine*) NEAR/2 (abuse OR depend* OR withdrawal))  methadone  addict*  psychoactive*  'drug withdrawal'  'withdrawal syndrome'  'alcoholic patient*'  'alcoholic subject*'  alcoholism  'alcohol depend*'  'alcohol-related disorder*'  'chronic ethanol*'  'chronic alcohol*'  'alcohol withdrawal'  'ethanol withdrawal'  schizophren*  schizotyp*  schizoaffective  ((delusional OR paranoid) NEAR/2 disorder*)  hallucination*  psychotic  schizoaffective  psychosis  psychoses  ((manic OR bipolar OR mood) NEAR/2 disorder*)  mania*  hypomania*  (suicide NEAR/1 attempt*)  suicidal*  'post-traumatic stress'  'posttraumatic stress'  'stress disorder*'  epileps*  "seizure disorder*"  aura  auras |

|  | **Concept: Intervention Study** |
| --- | --- |
| Subject Headings  (Emtree) | 'clinical trial'/exp  'comparative study'/exp  'evaluation study'/exp  'crossover procedure'/exp  'clinical trial (topic)'/exp  'intervention study'/exp  'experimental study'/exp |
| Free text terms  (searched in title, abstract, and keyword (:ti,ab,kw)) | random*  controll*  'intervention study'  'experimental study'  'comparative study'  trial  trials  evaluat*  repeat*  compar*  versus  'before and after'  'interrupted time series' |
|  | NOT ('animal'/exp NOT ('animal'/exp AND 'human'/exp)) |

('integrated health care system'/exp OR 'collaborative care team'/exp OR 'integrated care':jt OR ('integrated care' OR 'shared care' OR 'collaborative care' OR 'collaborative manag*' OR ((Integrat* OR coordinat* OR collaborat* OR co-locat* OR comanag* OR co-manag*) AND care AND (health OR healthcare) AND (services OR delivery OR manag* OR systems OR model* OR organisational OR organizational OR quality)) OR (('disease management' or 'case management') AND (care OR health OR healthcare) AND (services OR delivery OR model* OR quality))):ti,ab,kw)

**AND**

('mental disease'/exp OR 'mood disorder'/exp OR 'anxiety disorder'/exp OR 'dissociative disorder'/exp OR 'psychosis'/exp OR 'drug dependence'/exp OR 'cocaine smoking'/exp OR 'methadone'/exp OR 'withdrawal syndrome'/exp OR 'mania'/exp OR 'suicide attempt'/exp OR 'suicidal ideation'/exp OR 'epilepsy'/exp OR (depress* OR anxiet* OR ((serious OR severe OR major) NEXT/1 mental NEXT/1 (disorder* OR illness* OR condition* OR disease*)) OR 'drug abuse' OR 'drug depend*' OR 'substance abuse' OR 'substance-related disorder*' OR 'substance use disorder*' OR 'opioid use disorder*' OR oud OR ((cocaine OR heroin OR morphine*) NEAR/2 (abuse OR depend* OR withdrawal)) OR methadone OR addict* OR psychoactive* OR 'drug withdrawal' OR 'withdrawal syndrome' OR 'alcoholic patient*' OR 'alcoholic subject*' OR alcoholism OR 'alcohol depend*' OR 'alcohol-related disorder*' OR 'chronic ethanol*' OR 'chronic alcohol*' OR 'alcohol withdrawal' OR 'ethanol withdrawal' OR schizophren* OR schizotyp* OR schizoaffective OR ((delusional OR paranoid) NEAR/2 disorder*) OR hallucination* OR psychotic OR schizoaffective OR psychosis OR psychoses OR ((manic OR bipolar OR mood) NEAR/2 disorder*) OR mania* OR hypomania* OR (suicide NEAR/1 attempt*) OR suicidal* OR 'post-traumatic stress' OR 'posttraumatic stress' OR 'stress disorder*' OR epileps* OR 'seizure disorder*' OR aura OR auras):ti,ab,kw)

**AND**

('clinical trial'/exp OR 'comparative study'/exp OR 'evaluation study'/exp OR 'crossover procedure'/exp OR 'clinical trial (topic)'/exp OR 'intervention study'/exp OR 'experimental study'/exp OR (random* OR controll* OR 'intervention study' OR 'experimental study' OR 'comparative study' OR trial OR trials OR evaluat* OR repeat* OR compar* OR versus OR 'before and after' OR 'interrupted time series'):ti,ab)

**AND**

(

Afghanistan/exp OR Albania/exp OR Algeria/exp OR 'American Samoa'/exp OR Angola/exp OR 'Antigua and Barbuda'/exp OR Argentina/exp OR Armenia/exp OR Botswana/exp OR Azerbaijan/exp OR Bahrain/exp OR Balkan Peninsula/exp OR Baltic States/exp OR Bangladesh/exp OR Barbados/exp OR Belarus/exp OR Belize/exp OR Benin/exp OR Bhutan/exp OR Bolivia/exp OR 'Bosnia and Herzegovina'/exp OR Botswana/exp OR Brazil/exp OR Bulgaria/exp OR 'Burkina Faso'/exp OR Burundi/exp OR 'Cape Verde'/exp OR Cambodia/exp OR Cameroon/exp OR 'Central African Republic'/exp OR Chad/exp OR Chile/exp OR China/exp OR Colombia/exp OR Comoros/exp OR 'Democratic Republic Congo'/exp OR Congo/exp OR 'Costa Rica'/exp OR 'Cote d`Ivoire'/exp OR Croatia/exp OR Cuba/exp OR Cyprus/exp OR Czech Republic/exp OR Djibouti/exp OR Dominica/exp OR 'Dominican Republic'/exp OR Ecuador/exp OR Egypt/exp OR El Salvador/exp OR 'Equatorial Guinea'/exp OR Eritrea/exp OR Estonia/exp OR Eswatini/exp OR Ethiopia/exp OR Fiji/exp OR Gabon/exp OR Gambia/exp OR 'Georgia (republic)'/exp OR Ghana/exp OR Gibraltar/exp OR Greece/exp OR Grenada/exp OR Guam/exp OR Guatemala/exp OR Guinea/exp OR 'Guinea-Bissau'/exp OR Guyana/exp OR Haiti/exp OR Honduras/exp OR Hungary/exp OR India/exp OR Indonesia/exp OR Iran/exp OR Iraq/exp OR 'Isle of Man'/exp OR Jamaica/exp OR Jordan/exp OR Kazakhstan/exp OR Kenya/exp OR 'North Korea'/exp OR 'South Korea'/exp OR Kosovo/exp OR Kyrgyzstan/exp OR Laos/exp OR Latvia/exp OR Lebanon/exp OR Lesotho/exp OR Liberia/exp OR 'Libyan Arab Jamahiriya'/exp OR Lithuania/exp OR Macao/exp OR 'Republic of North Macedonia'/exp OR Madagascar/exp OR Malawi/exp OR Malaysia/exp OR Mali/exp OR Malta/exp OR Mayotte/exp OR 'Federated States of Micronesia'/exp OR Palau/exp OR Mauritania/exp OR Mauritius/exp OR Mexico/exp OR Moldova/exp OR Mongolia/exp OR 'Montenegro (republic)'/exp OR Morocco/exp OR Mozambique/exp OR Myanmar/exp OR Namibia/exp OR Nepal/exp OR 'Netherlands Antilles'/exp OR Nicaragua/exp OR Niger/exp OR Nigeria/exp OR Oman/exp OR Pakistan/exp OR Palestine/exp OR Panama/exp OR 'Papua New Guinea'/exp OR Paraguay/exp OR Peru/exp OR Philippines/exp OR Poland/exp OR Portugal/exp OR 'Puerto Rico'/exp OR Romania/exp OR 'Russian Federation'/exp OR Rwanda/exp OR Samoa/exp OR 'Sao Tome and Principe'/exp OR 'Saudi Arabia'/exp OR Senegal/exp OR Serbia/exp OR Seychelles/exp OR 'Sierra Leone'/exp OR Slovakia/exp OR Slovenia/exp OR Melanesia/exp OR Somalia/exp OR 'South Africa'/exp OR 'South Sudan'/exp OR Sri Lanka/exp OR 'Saint Kitts and Nevis'/exp OR 'Saint Lucia'/exp OR 'Saint Vincent and the Grenadines'/exp OR Sudan/exp OR Suriname/exp OR Syrian Arab Republic/exp OR Tajikistan/exp OR Tanzania/exp OR Thailand/exp OR 'Timor-Leste'/exp OR Togo/exp OR Tonga/exp OR 'Trinidad and Tobago'/exp OR Tunisia/exp OR Turkey/exp OR Turkmenistan/exp OR Uganda/exp OR Ukraine/exp OR Uruguay/exp OR Uzbekistan/exp OR Vanuatu/exp OR Venezuela/exp OR 'Viet Nam'/exp OR Yemen/exp OR Yugoslavia/exp OR Zambia/exp OR Zimbabwe/exp OR 'Africa south of the Sahara'/exp OR Sahel/exp OR 'Western Sahara'/exp OR 'Central Africa'/exp OR 'North Africa'/exp OR 'Indian Ocean'/exp OR 'Caribbean Islands'/exp OR Caribbean/exp OR 'Caribbean Netherlands'/exp OR 'Pacific islands'/exp OR 'Central America'/exp OR 'South and Central America'/exp OR 'South America'/exp OR 'Middle East'/exp OR 'Central Asia'/exp OR 'Northern Asia'/exp OR 'Southeast Asia'/exp OR 'Western Asia'/de OR 'Eastern Europe'/de OR 'developing country'/exp OR 'low income country'/exp OR 'middle income country'/exp OR

African/exp OR 'Central Asian'/exp OR 'South Asian'/exp OR 'Southeast Asian'/exp OR 'West Asian'/exp OR Chinese/exp OR 'North Korean'/exp OR 'South Korean'/exp OR 'Caribbean (person)'/exp OR 'Central American'/exp OR 'Melanesian'/exp OR 'Micronesian'/exp OR 'South American'/exp OR 'Eastern European'/exp OR

(Afghan* OR Albania* OR Algeria OR 'American Samoa*' OR Angola* OR Antigua* OR Barbuda* OR Argentin* OR Armenia* OR Aruba* OR Azerbaijan* OR Bahrain* OR Bangladesh* OR Bangalees OR Bajan* OR Barbados OR Belarus* OR Byelarus* OR Belorussia* OR Byelorussia* OR Belize* OR 'British Honduras' OR Benin* OR Dahomey OR Bhutan* OR Bolivia* OR Bosnia* OR Hercegovina* OR Herzegovina* OR Botswana* OR Batswana OR Brazil* OR Brasil* OR Bulgaria OR 'Burkina Faso' OR 'Burkina Fasso' OR Burkinabe OR Burkinese OR 'Upper Volta' OR Burundi* OR Urundi OR 'Cabo Verde*' OR 'Cape Verde*' OR Cambodia* OR Kampuchea OR Khmer OR Cameroon* OR Cameron* OR Cameroun* OR 'Central African' OR 'Ubangi Shari' OR Chad OR Chadian* OR Chile* OR China OR Chinese OR Colombia* OR Comoros OR Comores OR 'Comoro Islands' OR Comorian* OR Mayotte OR Congo OR Congolese OR Zaire OR 'Costa Rica*' OR 'Cote D`ivoire' OR 'Cote D` Ivoire' OR 'Cote Divoire' OR 'Cote D Ivoire' OR 'Ivory Coast' OR Ivorian* OR Croatia* OR Cuba OR Cuban* OR Cyprus OR Cypriot* OR Czech* OR Djibouti* OR 'French Somaliland' OR Dominica* OR Ecuador* OR Egypt* OR 'United Arab Republic' OR 'El Salvador' OR Salvadoran* OR 'Equatorial Guinea*' OR Equatoguinean* OR 'Spanish Guinea' OR Eritrea* OR Estonia* OR Eswatini OR Swaziland OR Swazi OR Swazis OR Swati OR Swatis OR Ethiopia* OR Fiji* OR Gabon* OR Gambia* OR Georgia* OR Ghana* OR 'Gold Coast' OR Gibraltar* OR Greece OR Greek* OR Grenada* OR Guam OR Guamanian* OR Guatemala OR Guatemalan* OR Guinea* OR Guyan* OR Guian* OR Haiti* OR Hispaniola OR Hondura* OR Hungar* OR Indian OR Indians OR India OR Indonesia* OR Timor* OR Iran* OR Iraq* OR 'Isle Of Man' OR Manx OR Jamaica* OR Jordan* OR Kazakh* OR Kenya* OR Korea* OR Kosovo* OR Kosovar* OR Kyrgyz* OR Kirghiz* OR Lao OR Laos OR Laotian* OR Latvia* OR Lebanon OR Lebanese OR Lesoth* OR Basutoland OR Mosotho OR Basotho OR Liberia* OR Libya* OR Lithuania* OR Macau OR Macao OR Macanese OR Macedonia* OR Madagasca* OR 'Malagasy Republic' OR Malawi* OR Nyasaland OR Malay* OR Maldiv* OR 'Indian Ocean' OR Mali OR Malian* OR Malta OR Maltese OR Micronesia* OR Kirabati* OR 'Marshall Islands' OR Marshallese OR Nauru* OR 'Northern Mariana Islands' OR Palau* OR Tuvalu* OR Mauritania OR Mauritius OR Mauritanian* OR Mauritian* OR Mexic* OR Moldov* OR Mongol* OR Montenegr* OR Morocc* OR Ifni OR Mozambique OR Mozambican* OR 'Portuguese East Africa' OR Myanma* OR Burma OR Burmese OR Namibia* OR Nepal* OR 'Netherlands Antille*' OR Nicaragua* OR Niger* OR Mariana* OR Oman OR Omani OR Muscat OR Pakistan* OR Palestin* OR Panama* OR 'Papua New Guinea*' OR Paraguay* OR Peru OR Peruvian* OR Philipine* OR Philippine* OR Phillipine* OR Phillippine* OR Filipino* OR Filipina* OR Poland OR Polish OR Portug* OR 'Puerto Ric*' OR Romania* OR Russia* OR Ussr OR Soviet* OR Rwand* OR Ruand* OR Samoa* OR 'Pacific Islands' OR Polynesia OR 'Samoan Islands' OR 'Sao Tome And Principe' OR 'Sao Tomean*' OR Santomean* OR Saudi* OR Senegal* OR Serbia* OR Seychell* OR 'Sierra Leone*' OR Slovak* OR Slovenia* OR Melanesia* OR 'Solomon Island*' OR 'Norfolk Island*' OR Somali* OR 'South Africa*' OR 'South Sudan*' OR 'Sri Lanka*' OR Ceylon* OR 'Saint Kitts And Nevis' OR 'St Kitts And Nevis' OR Kittitian* OR Nevisian* OR 'Saint Lucia*' OR 'St Lucia' OR 'Saint Vincent' OR 'St Vincent' OR Vincentian* OR Grenadines OR Sudan OR Sudanese OR Surinam* OR Syria OR Syrian* OR Tajik* OR Tadjik* OR Tadzhik* OR Tanzania* OR Tanganyika* OR Thai OR Thailand OR Siam OR 'Timor Leste' OR 'East Timor' OR Timorese* OR Togo OR Togolese OR Tonga OR Tongan* OR Trinidad* OR Tobago* OR Tunisia* OR Turkey OR Turkmen* OR Uganda* OR Ukrain* OR Uruguay* OR Uzbek* OR Vanuatu* OR 'New Hebride*' OR Venezuela* OR Vietnam* OR 'Viet Nam' OR 'Middle East' OR 'West Bank' OR Gaza OR Yemen* OR Yugoslav* OR Zambia* OR Zimbabwe* OR 'Northern Rhodesia*'):ti,ab,kw OR

('global south' OR 'Africa south of the Sahara' OR 'Saharan Africa' OR 'subSaharan Africa' OR 'central Africa' OR 'north Africa' OR 'northern Africa' OR Magreb OR Maghreb OR Sahara OR 'southern Africa' OR 'east Africa' OR 'eastern Africa' OR 'west Africa' OR 'western Africa' OR 'West Indies' OR 'Indian Ocean islands' OR Caribbean OR 'Central America' OR 'Latin America' OR 'South America' OR 'Central Asia' OR 'north Asia' OR 'northern Asia' OR 'southeastern Asia' OR 'south eastern Asia' OR 'southeast Asia' OR 'south east Asia' OR 'western Asia' OR 'east Europe' OR 'eastern Europe'):ti,ab,kw OR

(LMIC OR LMICS OR ((developing OR 'less developed' OR 'lesser developed' OR 'under developed' OR underdeveloped OR 'middle income' OR 'low income' OR 'lower income' OR underserved OR deprived OR poor OR poorer OR lami OR transitional) NEXT/1 countries) OR ((developing OR 'less developed' OR 'under developed' OR underdeveloped OR 'middle income' OR 'low income' OR 'lower income' OR poor OR lami OR transitional) NEXT/1 country) OR ((developing OR 'less developed' OR underdeveloped OR 'middle income' OR 'low income' OR poor OR emerging) NEXT/1 nation) OR ((developing OR 'less developed' OR 'lesser developed' OR 'under developed' OR underdeveloped OR 'middle income' OR 'low income' OR 'lower income' OR underserved OR poor OR poorer OR emerging) NEXT/1 nations) OR ((developing OR underdeveloped OR 'middle income' OR 'low income' OR 'lower income' OR underserved OR 'under served' OR deprived OR poor OR poorer) NEXT/1 (population OR populations)) OR ((developing OR 'less developed' OR 'under developed' OR underdeveloped OR poor OR third) NEXT/1 world) OR ((developing OR 'less developed' OR underdeveloped OR 'middle income' OR 'low income' OR 'lower income' OR emerging) NEXT/1 economies) OR ((developing OR 'less developed' OR 'middle income' OR 'low income') NEXT/1 economy) OR 'low gdp' OR 'low gnp' OR 'low gross domestic' OR 'low gross national' OR 'lower gdp' OR 'lower gross domestic'):ti,ab,kw

)

NOT ('child'/exp NOT ('child'/exp AND 'adult'/exp))

1,619 results as of 5/23/22

**CENTRAL (Wiley Cochrane Library)**

|  | **Concept: Integrated Care** |
| --- | --- |
| Subject Headings  [mh] | [mh "Delivery of Health Care, Integrated"] |
| Free text terms  (searched in journal title (:so) | [so "integrated care"] |
| Free text terms  (searched in title, abstract, and keyword (:ti,ab,kw)) | ”integrated care”  ”shared care”  ”collaborative care”  ”collaborative manag*”  ((Integrat* OR coordinat* OR collaborat* OR co-locat* OR comanag* OR co-manag*) AND care AND (health OR healthcare) AND (services OR delivery OR manag* OR systems OR model* OR organisational OR organizational OR quality))  ((”disease management” OR ”case management”) AND (care OR health OR healthcare) AND (services OR delivery OR model* OR quality)) |

|  | **Concept: Integrated Care** |
| --- | --- |
| Subject Headings  [mh] | [mh "Mental Disorders"]  [mh "Mood Disorders"]  [mh "Anxiety Disorders"]  [mh "Bipolar and Related Disorders"]  [mh "dissociative disorders"]  [mh "Schizophrenia Spectrum and Other Psychotic Disorders"]  [mh "Trauma and Stressor Related Disorders"]  [mh "Substance-Related Disorders"]  [mh "Cocaine Smoking"]  [mh Methadone]  [mh Hallucinations]  [mh Mania]  [mh "Suicide, Attempted"]  [mh "Suicidal Ideation"]  [mh "Epilepsy"] |
| Free text terms  (searched in title, abstract, and keyword (:ti,ab,kw)) | depress*  anxiet*  ((serious OR severe OR major) NEXT mental NEXT (disorder* OR illness* OR condition* OR disease*))  "drug abuse"  (drug NEXT depend*)  "substance abuse"  (("substance-related" OR "substance use" OR "opioid use" OR "alcohol-related") NEXT disorder*)  oud  ((cocaine OR heroin OR morphine*) NEAR/1 (abuse OR depend* OR withdrawal))  methadone  addict*  psychoactive*  "drug withdrawal"  "withdrawal syndrome"  (alcoholic NEXT (patient* OR subject*))  alcoholism  (alcohol NEXT depend*)  (chronic NEXT (ethanol* OR alcohol*))  "alcohol withdrawal"  "ethanol withdrawal"  schizophren*  schizotyp*  schizoaffective  ((delusional OR paranoid) NEAR/1 disorder*)  hallucination*  psychotic  schizoaffective  psychosis  psychoses  ((manic OR bipolar OR mood) NEAR/1 disorder*)  mania*  hypomania*  (suicide NEAR/1 attempt*)  suicidal*  "post-traumatic stress"  "posttraumatic stress"  (stress NEXT disorder*)  epileps*  (seizure NEXT disorder*)  aura  auras |

| **Limits** | NOT ([mh "Animals"] NOT ([mh "Animals"] AND [mh "Humans"]))  NOT ([mh "child"] NOT ([mh "child"] AND [mh "adult"])) |
| --- | --- |

| ID | Search | Hits |
| --- | --- | --- |
| #1 | [mh "Delivery of Health Care, Integrated"] | 428 |
| #2 | "integrated care":so | 5 |
| #3 | ("integrated care" OR "shared care" OR "collaborative care" OR (collaborative* NEXT manag*) OR ((Integrat* OR coordinat* OR collaborat* OR co-locat* OR comanage* OR co-manage*) AND care AND (health OR healthcare) AND (services OR delivery OR manag* OR systems OR model* OR organisational OR organizational OR quality)) OR ((Disease management OR Case management) AND (care OR health OR healthcare) AND (services OR delivery OR model* OR quality))):ti,ab,kw | 28935 |
| #4 | #1 OR #2 OR #3 | 28938 |
| #5 | [mh "Mental Disorders"] OR [mh "Mood Disorders"] OR [mh "Anxiety Disorders"] OR [mh "Bipolar and Related Disorders"] OR [mh "dissociative disorders"] OR [mh "Schizophrenia Spectrum and Other Psychotic Disorders"] OR [mh "Trauma and Stressor Related Disorders"] OR [mh "Substance-Related Disorders"] OR [mh "Cocaine Smoking"] OR [mh Methadone] OR [mh Hallucinations] OR [mh Mania] OR [mh "Suicide, Attempted"] OR [mh "Suicidal Ideation"] OR [mh "Epilepsy"] | 84695 |
| #6 | (depress* OR anxiety* OR ((serious OR severe OR major) NEXT mental NEXT (disorder* OR illness* OR condition* OR disease*)) OR "drug abuse" OR (drug NEXT depend*) OR "substance abuse" OR (("substance-related" OR "substance use" OR "opioid use" OR "alcohol-related") NEXT disorder*) OR oud OR ((cocaine OR heroin OR morphine*) NEAR/2 (abuse OR depend* OR withdrawal)) OR methadone OR addict* OR psychoactive* OR "drug withdrawal" OR "withdrawal syndrome" OR (alcoholic NEXT (patient* OR subject*)) OR alcoholism OR (alcohol NEXT depend*) OR (chronic NEXT (ethanol* OR alcohol*)) OR "alcohol withdrawal" OR "ethanol withdrawal" OR schizophren* OR schizotyp* OR schizoaffective OR ((delusional OR paranoid) NEAR/1 disorder*) OR hallucination* OR psychotic OR schizoaffective OR psychosis OR psychoses OR ((manic OR bipolar OR mood) NEAR/1 disorder*) OR mania* OR hypomania* OR (suicide NEAR/1 attempt*) OR suicidal* OR "post-traumatic stress" OR "posttraumatic stress" OR (stress NEXT disorder*) OR epileps* OR (seizure NEXT disorder*) OR aura OR auras):ti,ab,kw | 202725 |
| #7 | #5 OR #6 | 232964 |
| #8 | [mh "Afghanistan"] OR [mh "Albania"] OR [mh "Algeria"] OR [mh "American Samoa"] OR [mh "Angola"] OR [mh "Antigua and Barbuda"] OR [mh "Argentina"] OR [mh "Armenia"] OR [mh "Aruba"] OR [mh "Azerbaijan"] OR [mh "Bahrain"] OR [mh "Bangladesh"] OR [mh "Barbados"] OR [mh "Republic of Belarus"] OR [mh "Belize"] OR [mh "Benin"] OR [mh "Bhutan"] OR [mh "Bolivia"] OR [mh "Bosnia and Herzegovina"] OR [mh "Botswana"] OR [mh "Brazil"] OR [mh "Bulgaria"] OR [mh "Burkina Faso"] OR [mh "Burundi"] OR [mh "Cabo Verde"] OR [mh "Cambodia"] OR [mh "Cameroon"] OR [mh "Central African Republic"] OR [mh "Chad"] OR [mh "Chile"] OR [mh "China"] OR [mh "Colombia"] OR [mh "Comoros"] OR [mh "Democratic Republic of The Congo"] OR [mh "Congo"] OR [mh "Costa Rica"] OR [mh "Cote D’ivoire"] OR [mh "Croatia"] OR [mh "Cuba"] OR [mh "Cyprus"] OR [mh "Czech Republic"] OR [mh "Djibouti"] OR [mh "Dominica"] OR [mh "Dominican Republic"] OR [mh "Ecuador"] OR [mh "Egypt"] OR [mh "El Salvador"] OR [mh "Equatorial Guinea"] OR [mh "Eritrea"] OR [mh "Estonia"] OR [mh "Swaziland"] OR [mh "Ethiopia"] OR [mh "Fiji"] OR [mh "Gabon"] OR [mh "Gambia"] OR [mh "Georgia (Republic)"] OR [mh "Ghana"] OR [mh "Gibraltar"] OR [mh "Greece"] OR [mh "Grenada"] OR [mh "Guam"] OR [mh "Guatemala"] OR [mh "Guinea"] OR [mh "Guinea Bissau"] OR [mh "Guyana"] OR [mh "Haiti"] OR [mh "Honduras"] OR [mh "Hungary"] OR [mh "India"] OR [mh "Indonesia"] OR [mh "Iran"] OR [mh "Iraq"] OR [mh "Jamaica"] OR [mh "Jordan"] OR [mh "Kazakhstan"] OR [mh "Kenya"] OR [mh "Democratic People’s Republic of Korea"] OR [mh "Republic of Korea"] OR [mh "Kosovo"] OR [mh "Kyrgyzstan"] OR [mh "Laos"] OR [mh "Latvia"] OR [mh "Lebanon"] OR [mh "Lesotho"] OR [mh "Liberia"] OR [mh "Libya"] OR [mh "Lithuania"] OR [mh "Macau"] OR [mh "Republic of North Macedonia"] OR [mh "Madagascar"] OR [mh "Malawi"] OR [mh "Malaysia"] OR [mh "Indian Ocean Islands"] OR [mh "Mali"] OR [mh "Malta"] OR [mh "Micronesia"] OR [mh "Palau"] OR [mh "Mauritania"] OR [mh "Mauritius"] OR [mh "Mexico"] OR [mh "Moldova"] OR [mh "Mongolia"] OR [mh "Montenegro"] OR [mh "Morocco"] OR [mh "Mozambique"] OR [mh "Myanmar"] OR [mh "Namibia"] OR [mh "Nepal"] OR [mh "Netherlands Antilles"] OR [mh "Nicaragua"] OR [mh "Niger"] OR [mh "Nigeria"] OR [mh "Oman"] OR [mh "Pakistan"] OR [mh "Panama"] OR [mh "Papua New Guinea"] OR [mh "Paraguay"] OR [mh "Peru"] OR [mh "Philippines"] OR [mh "Poland"] OR [mh "Portugal"] OR [mh "Puerto Rico"] OR [mh "Romania"] OR [mh "Russia"] OR [mh "Rwanda"] OR [mh "Samoa"] OR [mh "Sao Tome and Principe"] OR [mh "Saudi Arabia"] OR [mh "Senegal"] OR [mh "Serbia"] OR [mh "Seychelles"] OR [mh "Sierra Leone"] OR [mh "Slovakia"] OR [mh "Slovenia"] OR [mh "Melanesia"] OR [mh "Somalia"] OR [mh "South Africa"] OR [mh "South Sudan"] OR [mh "Sri Lanka"] OR [mh "Saint Kitts and Nevis"] OR [mh "Saint Lucia"] OR [mh "Saint Vincent and The Grenadines"] OR [mh "Sudan"] OR [mh "Suriname"] OR [mh "Syria"] OR [mh "Tajikistan"] OR [mh "Tanzania"] OR [mh "Thailand"] OR [mh "Timor Leste"] OR [mh "Togo"] OR [mh "Tonga"] OR [mh "Trinidad and Tobago"] OR [mh "Tunisia"] OR [mh "Turkey"] OR [mh "Turkmenistan"] OR [mh "Uganda"] OR [mh "Ukraine"] OR [mh "Uruguay"] OR [mh "Uzbekistan"] OR [mh "Vanuatu"] OR [mh "Venezuela"] OR [mh "Vietnam"] OR [mh "Middle East"] OR [mh "Yemen"] OR [mh "Yugoslavia"] OR [mh "Zambia"] OR [mh "Zimbabwe"] OR [mh "Africa South of The Sahara"] OR [mh "Africa, Central"] OR [mh "Africa, Northern"] OR [mh "Africa, Southern"] OR [mh "Africa, Eastern"] OR [mh "Africa, Western"] OR [mh "West Indies"] OR [mh "Indian Ocean Islands"] OR [mh "Caribbean Region"] OR [mh "Central America"] OR [mh "Latin America"] OR [mh "South America"] OR [mh "Asia, Central"] OR [mh "Asia, Northern"] OR [mh "Asia, Southeastern"] OR [mh "Asia, Western"] OR [mh "Europe, Eastern"] OR [mh "Developing Countries"] | 31787 |
| #9 | (Afghan* OR Albania* OR Algeria OR "American Samoa" OR "American Samoan" OR "American Samoans" OR Angola* OR Antigua* OR Barbuda* OR Argentin* OR Armenia* OR Aruba* OR Azerbaijan* OR Bahrain* OR Bangladesh* OR Bangalees OR Bajan* OR Barbados OR Belarus* OR Byelarus* OR Belorussia* OR Byelorussia* OR Belize* OR "British Honduras" OR Benin* OR Dahomey OR Bhutan* OR Bolivia* OR Bosnia* OR Hercegovina* OR Herzegovina* OR Botswana* OR Batswana OR Brazil* OR Brasil* OR Bulgaria OR "Burkina Faso" OR "Burkina Fasso" OR Burkinabe OR Burkinese OR "Upper Volta" OR Burundi* OR Urundi OR "Cabo Verde" OR "Cape Verde" OR "Cape Verdean" OR "Cape Verdeans" OR "Cabo Verdean" OR "Cabo Verdeans" OR Cambodia* OR Kampuchea OR Khmer OR Cameroon* OR Cameron* OR Cameroun* OR "Central African" OR "Ubangi Shari" OR Chad OR Chadian* OR Chile* OR China OR Chinese OR Colombia* OR Comoros OR Comores OR "Comoro Islands" OR Comorian* OR Mayotte OR Congo OR Congolese OR Zaire OR "Costa Rica" OR "Costa Rican" OR "Costa Ricans" OR "Cote D’ivoire" OR "Cote D’ Ivoire" OR "Cote Divoire" OR "Cote D Ivoire" OR "Ivory Coast" OR Ivorian* OR Croatia* OR Cuba OR Cuban* OR Cyprus OR Cypriot* OR Czech* OR Djibouti* OR "French Somaliland" OR Dominica* OR Ecuador* OR Egypt* OR "United Arab Republic" OR "El Salvador" OR Salvadoran* OR "Equatorial Guinea" OR "Equatorial Guinean" OR "Equatorial Guineans" OR Equatoguinean* OR "Spanish Guinea" OR Eritrea* OR Estonia* OR Eswatini OR Swaziland OR Swazi OR Swazis OR Swati OR Swatis OR Ethiopia* OR Fiji* OR Gabon* OR Gambia* OR Georgia* OR Ghana* OR "Gold Coast" OR Gibraltar* OR Greece OR Greek* OR Grenada* OR Guam OR Guamanian* OR Guatemala OR Guatemalan* OR Guinea* OR Guyan* OR Guian* OR Haiti* OR Hispaniola OR Hondura* OR Hungar* OR Indian OR Indians OR India OR Indonesia* OR Timor* OR Iran* OR Iraq* OR "Isle Of Man" OR Manx OR Jamaica* OR Jordan* OR Kazakh* OR Kenya* OR Korea* OR Kosovo* OR Kosovar* OR Kyrgyz* OR Kirghiz* OR Lao OR Laos OR Laotian* OR Latvia* OR Lebanon OR Lebanese OR Lesoth* OR Basutoland OR Mosotho OR Basotho OR Liberia* OR Libya* OR Lithuania* OR Macau OR Macao OR Macanese OR Macedonia* OR Madagasca* OR "Malagasy Republic" OR Malawi* OR Nyasaland OR Malay* OR Maldiv* OR "Indian Ocean" OR Mali OR Malian* OR Malta OR Maltese OR Micronesia* OR Kirabati* OR "Marshall Islands" OR Marshallese OR Nauru* OR "Northern Mariana Islands" OR Palau* OR Tuvalu* OR Mauritania OR Mauritius OR Mauritanian* OR Mauritian* OR Mexic* OR Moldov* OR Mongol* OR Montenegr* OR Morocc* OR Ifni OR Mozambique OR Mozambican* OR "Portuguese East Africa" OR Myanma* OR Burma OR Burmese OR Namibia* OR Nepal* OR "Netherlands Antilles" OR "Netherlands Antillean" OR "Netherlands Antilleans" OR Nicaragua* OR Niger* OR Mariana* OR Oman OR Omani OR Muscat OR Pakistan* OR Palestin* OR Panama* OR "Papua New Guinea" OR "Papua New Guinean" OR "Papua New Guineans" OR Paraguay* OR Peru OR Peruvian* OR Philipine* OR Philippine* OR Phillipine* OR Phillippine* OR Filipino* OR Filipina* OR Poland OR Polish OR Portug* OR "Puerto Rico" OR "Puerto Rican" OR "Puerto Ricans" OR Romania* OR Russia* OR Ussr OR Soviet* OR Rwand* OR Ruand* OR Samoa* OR "Pacific Islands" OR Polynesia OR "Samoan Islands" OR "Sao Tome And Principe" OR "Sao Tomean" OR "Sao Tomeans" OR Santomean* OR Saudi* OR Senegal* OR Serbia* OR Seychell* OR "Sierra Leone*" OR "Sierra Leonean" OR "Sierra Leoneans" OR Slovak* OR Slovenia* OR Melanesia* OR ((Solomon OR Norfolk) NEXT Island*) OR Somali* OR "South Africa*" OR "South Sudan*" OR "Sri Lanka*" OR Ceylon* OR "Saint Kitts And Nevis" OR "St Kitts And Nevis" OR Kittitian* OR Nevisian* OR "Saint Lucia*" OR "St Lucia" OR "Saint Vincent" OR "St Vincent" OR Vincentian* OR Grenadines OR Sudan OR Sudanese OR Surinam* OR Syria OR Syrian* OR Tajik* OR Tadjik* OR Tadzhik* OR Tanzania* OR Tanganyika* OR Thai OR Thailand OR Siam OR "Timor Leste" OR "East Timor" OR Timorese* OR Togo OR Togolese OR Tonga OR Tongan* OR Trinidad* OR Tobago* OR Tunisia* OR Turkey OR Turkmen* OR Uganda* OR Ukrain* OR Uruguay* OR Uzbek* OR Vanuatu* OR "New Hebride*" OR Venezuela* OR Vietnam* OR "Viet Nam" OR "Middle East" OR "West Bank" OR Gaza OR Yemen* OR Yugoslav* OR Zambia* OR Zimbabwe* OR "Northern Rhodesia*"):ti,ab,kw | 143399 |
| #10 | ("global south" OR "Africa south of the Sahara" OR "Saharan Africa" OR "subSaharan Africa" OR "central Africa" OR "north Africa" OR "northern Africa" OR Magreb OR Maghreb OR Sahara OR "southern Africa" OR "east Africa" OR "eastern Africa" OR "west Africa" OR "western Africa" OR "West Indies" OR "Indian Ocean islands" OR Caribbean OR "Central America" OR "Latin America" OR "South America" OR "Central Asia" OR "north Asia" OR "northern Asia" OR "southeastern Asia" OR "south eastern Asia" OR "southeast Asia" OR "south east Asia" OR "western Asia" OR "east Europe" OR "eastern Europe"):ti,ab,kw | 5404 |
| #11 | (LMIC OR LMICS OR ((developing OR 'less developed' OR 'lesser developed' OR 'under developed' OR underdeveloped OR 'middle income' OR 'low income' OR 'lower income' OR underserved OR deprived OR poor OR poorer OR lami OR transitional) NEXT countries) OR ((developing OR 'less developed' OR 'under developed' OR underdeveloped OR 'middle income' OR 'low income' OR 'lower income' OR poor OR lami OR transitional) NEXT country) OR ((developing OR 'less developed' OR underdeveloped OR 'middle income' OR 'low income' OR poor OR emerging) NEXT nation) OR ((developing OR 'less developed' OR 'lesser developed' OR 'under developed' OR underdeveloped OR 'middle income' OR 'low income' OR 'lower income' OR underserved OR poor OR poorer OR emerging) NEXT nations) OR ((developing OR underdeveloped OR 'middle income' OR 'low income' OR 'lower income' OR underserved OR 'under served' OR deprived OR poor OR poorer) NEXT (population OR populations)) OR ((developing OR 'less developed' OR 'under developed' OR underdeveloped OR poor OR third) NEXT world) OR ((developing OR 'less developed' OR underdeveloped OR 'middle income' OR 'low income' OR 'lower income' OR emerging) NEXT economies) OR ((developing OR 'less developed' OR 'middle income' OR 'low income') NEXT economy) OR 'low gdp' OR 'low gnp' OR 'low gross domestic' OR 'low gross national' OR 'lower gdp' OR 'lower gross domestic'):ti,ab,kw | 9892 |
| #12 | #8 OR #9 OR #10 OR #11 | 150800 |
| #13 | #4 AND #7 AND #12 | 1096 |
| #14 | #13 NOT ([mh "Animals"] NOT ([mh "Animals"] AND [mh "Humans"])) NOT ([mh "child"] NOT ([mh "child"] AND [mh "adult"])) in Trials | 1031 |

1,031 results as of 5/23/22

**PsycInfo; EBSCO**

|  | **Concept:** |
| --- | --- |
| PsycInfo Subject Headings (DE) | DE "Integrated Services" |
| MeSH terms (MA) | "Delivery of Health Care, Integrated" |
| Free text terms  (searched in journal title (SO)) | SO "integrated care" |
| Free text terms  (searched in Title, Abstract, and Keywords) | "integrated care"  "shared care"  "collaborative care"  "collaborative manag*"  ((Integrat* OR coordinat* OR collaborat* OR co-locat* OR comanag* OR co-manag*) AND care AND (health OR healthcare) AND (services OR delivery OR manag* OR systems OR model* OR organisational OR organizational OR quality))  (("disease management" OR "case management") AND (care OR health OR healthcare) AND (services OR delivery OR model* OR quality)) |

|  | **Concept:** |
| --- | --- |
| PsycInfo Subject Headings (DE) | "Mental Disorders"  "Serious Mental Illness"  "Affective Disorders"  "Disruptive Mood Dysregulation Disorder"  "Major Depression"  "Anxiety Disorders"  "Bipolar Disorder"  "Bipolar I Disorder"  "Bipolar II Disorder"  "Cyclothymic Disorder"  "Dissociative Disorders"  "Depersonalization"  "Depersonalization/Derealization Disorder"  "Dissociative Amnesia"  "Dissociative Identity Disorder"  "Fugue Reaction"  "Psychosis"  "Acute Psychosis"  "Affective Psychosis"  "Alcoholic Psychosis"  "Capgras Syndrome"  "Chronic Psychosis"  "Experimental Psychosis"  "Hallucinosis"  "Paranoia Psychosis"  "Postpartum Psychosis"  "Reactive Psychosis"  "Senile Psychosis"  "Toxic Psychoses"  "Schizophrenia"  "Acute Schizophrenia"  "Catatonic Schizophrenia"  "Childhood Schizophrenia"  "Paranoid Schizophrenia"  "Process Schizophrenia"  "Schizoaffective Disorder"  "Schizophrenia Disorganized Type"  "Schizophreniform Disorder"  "Undifferentiated Schizophrenia"  "Stress and Trauma Related Disorders"  "Acute Stress Disorder"  "Posttraumatic Stress Disorder"  "Complex PTSD"  "DESNOS"  "Hallucinations"  "Auditory Hallucinations"  "Drug Induced Hallucinations"  "Hypnagogic Hallucinations"  "Visual Hallucinations"  "Mania"  "Hypomania"  "Suicidal Ideation"  "Attempted Suicide"  "Substance Use Disorder"  "Addiction"  "Alcohol Use Disorder"  "Cannabis Use Disorder"  "Drug Abuse"  "Drug Dependency"  "Inhalant Abuse"  "Opioid Use Disorder"  "Inhalant Abuse"  "Polydrug Abuse"  "Crack Cocaine"  "Methadone"  "Epilepsy"  "Epileptic Seizures" |
| MeSH terms (MA) | "Mental Disorders"  "Mood Disorders"  "Anxiety Disorders"  "Bipolar and Related Disorders"  "dissociative disorders"  "Schizophrenia Spectrum and Other Psychotic Disorders"  Schizophrenia  "Trauma and Stressor Related Disorders"  "Substance-Related Disorders"  "Substance Withdrawal Syndrome"  "Alcoholism"  "Cocaine Smoking"  Methadone  Hallucinations  Mania  "Suicide, Attempted"  "Suicidal Ideation"  "Epilepsy" |
| Free text terms  (searched in Title, Abstract, and Keywords) | depress*  anxiet*  ((serious OR severe OR major) W0 mental W0 (disorder* OR illness* OR condition* OR disease*))  "drug abuse"  "drug depend*"  "substance abuse"  "substance-related disorder*"  "substance use disorder*"  "opioid use disorder*"  oud  ((cocaine OR heroin OR morphine*) N1 (abuse OR depend* OR withdrawal))  methadone  addict*  psychoactive*  "drug withdrawal"  "withdrawal syndrome"  "alcoholic patient*"  "alcoholic subject*"  alcoholism  "alcohol depend*"  "alcohol-related disorder*"  "chronic ethanol*"  "chronic alcohol*"  "alcohol withdrawal"  "ethanol withdrawal"  schizophren*  schizotyp*  schizoaffective  ((delusional OR paranoid) N1 disorder*)  hallucination*  psychotic  schizoaffective  psychosis  psychoses  ((manic OR bipolar OR mood) N1 disorder*)  mania*  hypomania*  (suicide N0 attempt*)  suicidal*  "post-traumatic stress"  "posttraumatic stress"  "stress disorder*"  epileps*  "seizure disorder*"  aura  auras |

|  | **Concept:** |
| --- | --- |
| PsycInfo Subject Headings (DE) | "Clinical Trials"  "Randomized Controlled Trials"  "Randomized Clinical Trials" |
| MeSH terms (MA) | “Clinical Trial”  “Comparative Study”  “Evaluation study”  “Cross-Over Studies”  “Clinical Trials as Topic” |
| Free text terms  (searched in Title, Abstract, and Keywords) | random*  controll*  "intervention study"  "experimental study"  "comparative study"  trial  trials  evaluat*  repeat*  compar*  versus  "before and after"  "interrupted time series" |

(DE "Integrated Services" OR MA "Delivery of Health Care, Integrated" OR SO "integrated care" OR TI("integrated care" OR "shared care" OR "collaborative care" OR "collaborative manag*" OR ((Integrat* OR coordinat* OR collaborat* OR co-locat* OR comanag* OR co-manag*) AND care AND (health OR healthcare) AND (services OR delivery OR manag* OR systems OR model* OR organisational OR organizational OR quality)) OR (("disease management" OR "case management") AND (care OR health OR healthcare) AND (services OR delivery OR model* OR quality))) OR AB("integrated care" OR "shared care" OR "collaborative care" OR "collaborative manag*" OR ((Integrat* OR coordinat* OR collaborat* OR co-locat* OR comanag* OR co-manag*) AND care AND (health OR healthcare) AND (services OR delivery OR manag* OR systems OR model* OR organisational OR organizational OR quality)) OR (("disease management" OR "case management") AND (care OR health OR healthcare) AND (services OR delivery OR model* OR quality))) OR KW("integrated care" OR "shared care" OR "collaborative care" OR "collaborative manag*" OR ((Integrat* OR coordinat* OR collaborat* OR co-locat* OR comanag* OR co-manag*) AND care AND (health OR healthcare) AND (services OR delivery OR manag* OR systems OR model* OR organisational OR organizational OR quality)) OR (("disease management" OR "case management") AND (care OR health OR healthcare) AND (services OR delivery OR model* OR quality))))

**AND**

(DE("Mental Disorders" OR "Serious Mental Illness" OR "Affective Disorders" OR "Disruptive Mood Dysregulation Disorder" OR "Major Depression" OR "Anxiety Disorders" OR "Bipolar Disorder" OR "Bipolar I Disorder" OR "Bipolar II Disorder" OR "Cyclothymic Disorder" OR "Dissociative Disorders" OR "Depersonalization" OR "Depersonalization/Derealization Disorder" OR "Dissociative Amnesia" OR "Dissociative Identity Disorder" OR "Fugue Reaction" OR "Psychosis" OR "Acute Psychosis" OR "Affective Psychosis" OR "Alcoholic Psychosis" OR "Capgras Syndrome" OR "Chronic Psychosis" OR "Experimental Psychosis" OR "Hallucinosis" OR "Paranoia Psychosis" OR "Postpartum Psychosis" OR "Reactive Psychosis" OR "Senile Psychosis" OR "Toxic Psychoses" OR "Schizophrenia" OR "Acute Schizophrenia" OR "Catatonic Schizophrenia" OR "Childhood Schizophrenia" OR "Paranoid Schizophrenia" OR "Process Schizophrenia" OR "Schizoaffective Disorder" OR "Schizophrenia Disorganized Type" OR "Schizophreniform Disorder" OR "Undifferentiated Schizophrenia" OR "Stress and Trauma Related Disorders" OR "Acute Stress Disorder" OR "Posttraumatic Stress Disorder" OR "Complex PTSD" OR "DESNOS" OR "Hallucinations" OR "Auditory Hallucinations" OR "Drug Induced Hallucinations" OR "Hypnagogic Hallucinations" OR "Visual Hallucinations" OR "Mania" OR "Hypomania" OR "Suicidal Ideation" OR "Attempted Suicide" OR "Substance Use Disorder" OR "Addiction" OR "Alcohol Use Disorder" OR "Cannabis Use Disorder" OR "Drug Abuse" OR "Drug Dependency" OR "Inhalant Abuse" OR "Opioid Use Disorder" OR "Inhalant Abuse" OR "Polydrug Abuse" OR "Crack Cocaine" OR "Methadone" OR "Epilepsy" OR "Epileptic Seizures")

OR

MA("Mental Disorders" OR "Mood Disorders" OR "Anxiety Disorders" OR "Bipolar and Related Disorders" OR "dissociative disorders" OR "Schizophrenia Spectrum and Other Psychotic Disorders" OR Schizophrenia OR "Trauma and Stressor Related Disorders" OR "Substance-Related Disorders" OR "Substance Withdrawal Syndrome" OR "Alcoholism" OR "Cocaine Smoking" OR Methadone OR Hallucinations OR Mania OR "Suicide, Attempted" OR "Suicidal Ideation" OR "Epilepsy")

OR

TI(depress* OR anxiet* OR ((serious OR severe OR major) W0 mental W0 (disorder* OR illness* OR condition* OR disease*)) OR "drug abuse" OR "drug depend*" OR "substance abuse" OR "substance-related disorder*" OR "substance use disorder*" OR "opioid use disorder*" OR oud OR ((cocaine OR heroin OR morphine*) N1 (abuse OR depend* OR withdrawal)) OR methadone OR addict* OR psychoactive* OR "drug withdrawal" OR "withdrawal syndrome" OR "alcoholic patient*" OR "alcoholic subject*" OR alcoholism OR "alcohol depend*" OR "alcohol-related disorder*" OR "chronic ethanol*" OR "chronic alcohol*" OR "alcohol withdrawal" OR "ethanol withdrawal" OR schizophren* OR schizotyp* OR schizoaffective OR ((delusional OR paranoid) N1 disorder*) OR hallucination* OR psychotic OR schizoaffective OR psychosis OR psychoses OR ((manic OR bipolar OR mood) N1 disorder*) OR mania* OR hypomania* OR (suicide N0 attempt*) OR suicidal* OR "post-traumatic stress" OR "posttraumatic stress" OR "stress disorder*" OR epileps* OR "seizure disorder*" OR aura OR auras)

OR

AB(depress* OR anxiet* OR ((serious OR severe OR major) W0 mental W0 (disorder* OR illness* OR condition* OR disease*)) OR "drug abuse" OR "drug depend*" OR "substance abuse" OR "substance-related disorder*" OR "substance use disorder*" OR "opioid use disorder*" OR oud OR ((cocaine OR heroin OR morphine*) N1 (abuse OR depend* OR withdrawal)) OR methadone OR addict* OR psychoactive* OR "drug withdrawal" OR "withdrawal syndrome" OR "alcoholic patient*" OR "alcoholic subject*" OR alcoholism OR "alcohol depend*" OR "alcohol-related disorder*" OR "chronic ethanol*" OR "chronic alcohol*" OR "alcohol withdrawal" OR "ethanol withdrawal" OR schizophren* OR schizotyp* OR schizoaffective OR ((delusional OR paranoid) N1 disorder*) OR hallucination* OR psychotic OR schizoaffective OR psychosis OR psychoses OR ((manic OR bipolar OR mood) N1 disorder*) OR mania* OR hypomania* OR (suicide N0 attempt*) OR suicidal* OR "post-traumatic stress" OR "posttraumatic stress" OR "stress disorder*" OR epileps* OR "seizure disorder*" OR aura OR auras)

OR

KW(depress* OR anxiet* OR ((serious OR severe OR major) W0 mental W0 (disorder* OR illness* OR condition* OR disease*)) OR "drug abuse" OR "drug depend*" OR "substance abuse" OR "substance-related disorder*" OR "substance use disorder*" OR "opioid use disorder*" OR oud OR ((cocaine OR heroin OR morphine*) N1 (abuse OR depend* OR withdrawal)) OR methadone OR addict* OR psychoactive* OR "drug withdrawal" OR "withdrawal syndrome" OR "alcoholic patient*" OR "alcoholic subject*" OR alcoholism OR "alcohol depend*" OR "alcohol-related disorder*" OR "chronic ethanol*" OR "chronic alcohol*" OR "alcohol withdrawal" OR "ethanol withdrawal" OR schizophren* OR schizotyp* OR schizoaffective OR ((delusional OR paranoid) N1 disorder*) OR hallucination* OR psychotic OR schizoaffective OR psychosis OR psychoses OR ((manic OR bipolar OR mood) N1 disorder*) OR mania* OR hypomania* OR (suicide N0 attempt*) OR suicidal* OR "post-traumatic stress" OR "posttraumatic stress" OR "stress disorder*" OR epileps* OR "seizure disorder*" OR aura OR auras))

**AND**

(DE("Clinical Trials" OR "Randomized Controlled Trials" OR "Randomized Clinical Trials") OR MA(“Clinical Trial” OR “Comparative Study” OR “Evaluation study” OR “Cross-Over Studies” OR “Clinical Trials as Topic”) OR

TI(random* OR controll* OR "intervention study" OR "experimental study" OR "comparative study" OR trial OR trials OR evaluat* OR repeat* OR compar* OR versus OR "before and after" OR "interrupted time series")

OR

AB(random* OR controll* OR "intervention study" OR "experimental study" OR "comparative study" OR trial OR trials OR evaluat* OR repeat* OR compar* OR versus OR "before and after" OR "interrupted time series")

OR

KW(random* OR controll* OR "intervention study" OR "experimental study" OR "comparative study" OR trial OR trials OR evaluat* OR repeat* OR compar* OR versus OR "before and after" OR "interrupted time series"))

**AND**

(MA("Afghanistan" OR "Albania" OR "Algeria" OR "American Samoa" OR "Angola" OR "Antigua and Barbuda" OR "Argentina" OR "Armenia" OR "Aruba" OR "Azerbaijan" OR "Bahrain" OR "Bangladesh" OR "Barbados" OR "Republic of Belarus" OR "Belize" OR "Benin" OR "Bhutan" OR "Bolivia" OR "Bosnia and Herzegovina" OR "Botswana" OR "Brazil" OR "Bulgaria" OR "Burkina Faso" OR "Burundi" OR "Cabo Verde" OR "Cambodia" OR "Cameroon" OR "Central African Republic" OR "Chad" OR "Chile" OR "China" OR "Colombia" OR "Comoros" OR "Democratic Republic of The Congo" OR "Congo" OR "Costa Rica" OR "Cote D’ivoire" OR "Croatia" OR "Cuba" OR "Cyprus" OR "Czech Republic" OR "Djibouti" OR "Dominica" OR "Dominican Republic" OR "Ecuador" OR "Egypt" OR "El Salvador" OR "Equatorial Guinea" OR "Eritrea" OR "Estonia" OR "Swaziland" OR "Ethiopia" OR "Fiji" OR "Gabon" OR "Gambia" OR "Georgia (Republic)" OR "Ghana" OR "Gibraltar" OR "Greece" OR "Grenada" OR "Guam" OR "Guatemala" OR "Guinea" OR "Guinea Bissau" OR "Guyana" OR "Haiti" OR "Honduras" OR "Hungary" OR "India" OR "Indonesia" OR "Iran" OR "Iraq" OR "Jamaica" OR "Jordan" OR "Kazakhstan" OR "Kenya" OR "Democratic People’s Republic of Korea" OR "Republic of Korea" OR "Kosovo" OR "Kyrgyzstan" OR "Laos" OR "Latvia" OR "Lebanon" OR "Lesotho" OR "Liberia" OR "Libya" OR "Lithuania" OR "Macau" OR "Republic of North Macedonia" OR "Madagascar" OR "Malawi" OR "Malaysia" OR "Indian Ocean Islands" OR "Mali" OR "Malta" OR "Micronesia" OR "Palau" OR "Mauritania" OR "Mauritius" OR "Mexico" OR "Moldova" OR "Mongolia" OR "Montenegro" OR "Morocco" OR "Mozambique" OR "Myanmar" OR "Namibia" OR "Nepal" OR "Netherlands Antilles" OR "Nicaragua" OR "Niger" OR "Nigeria" OR "Oman" OR "Pakistan" OR "Panama" OR "Papua New Guinea" OR "Paraguay" OR "Peru" OR "Philippines" OR "Poland" OR "Portugal" OR "Puerto Rico" OR "Romania" OR "Russia" OR "Rwanda" OR "Samoa" OR "Sao Tome and Principe" OR "Saudi Arabia" OR "Senegal" OR "Serbia" OR "Seychelles" OR "Sierra Leone" OR "Slovakia" OR "Slovenia" OR "Melanesia" OR "Somalia" OR "South Africa" OR "South Sudan" OR "Sri Lanka" OR "Saint Kitts and Nevis" OR "Saint Lucia" OR "Saint Vincent and The Grenadines" OR "Sudan" OR "Suriname" OR "Syria" OR "Tajikistan" OR "Tanzania" OR "Thailand" OR "Timor Leste" OR "Togo" OR "Tonga" OR "Trinidad and Tobago" OR "Tunisia" OR "Turkey" OR "Turkmenistan" OR "Uganda" OR "Ukraine" OR "Uruguay" OR "Uzbekistan" OR "Vanuatu" OR "Venezuela" OR "Vietnam" OR "Middle East" OR "Yemen" OR "Yugoslavia" OR "Zambia" OR "Zimbabwe" OR "Africa South of The Sahara" OR "Africa, Central" OR "Africa, Northern" OR "Africa, Southern" OR "Africa, Eastern" OR "Africa, Western" OR "West Indies" OR "Indian Ocean Islands" OR "Caribbean Region" OR "Central America" OR "Latin America" OR "South America" OR "Asia, Central" OR "Asia, Northern" OR "Asia, Southeastern" OR "Asia, Western" OR "Europe, Eastern" OR "Developing Countries") OR

TI(Afghan* OR Albania* OR Algeria OR "American Samoa*" OR Angola* OR Antigua* OR Barbuda* OR Argentin* OR Armenia* OR Aruba* OR Azerbaijan* OR Bahrain* OR Bangladesh* OR Bangalees OR Bajan* OR Barbados OR Belarus* OR Byelarus* OR Belorussia* OR Byelorussia* OR Belize* OR British Honduras OR Benin* OR Dahomey OR Bhutan* OR Bolivia* OR Bosnia* OR Hercegovina* OR Herzegovina* OR Botswana* OR Batswana OR Brazil* OR Brasil* OR Bulgaria OR Burkina Faso OR Burkina Fasso OR Burkinabe OR Burkinese OR Upper Volta OR Burundi* OR Urundi OR Cabo Verde* OR Cape Verde* OR Cambodia* OR Kampuchea OR Khmer OR Cameroon* OR Cameron* OR Cameroun* OR "Central African" OR Ubangi Shari OR Chad OR Chadian* OR Chile* OR China OR Chinese OR Colombia* OR Comoros OR Comores OR Comoro Islands OR Comorian* OR Mayotte OR Congo OR Congolese OR Zaire OR Costa Rica* OR Cote D’ivoire OR Cote D’ Ivoire OR Cote Divoire OR Cote D Ivoire OR Ivory Coast OR Ivorian* OR Croatia* OR Cuba OR Cuban* OR Cyprus OR Cypriot* OR Czech* OR Djibouti* OR French Somaliland OR Dominica* OR Ecuador* OR Egypt* OR United Arab Republic OR El Salvador OR Salvadoran* OR "Equatorial Guinea*" OR Equatoguinean* OR Spanish Guinea OR Eritrea* OR Estonia* OR Eswatini OR Swaziland OR Swazi OR Swazis OR Swati OR Swatis OR Ethiopia* OR Fiji* OR Gabon* OR Gambia* OR Georgia* OR Ghana* OR Gold Coast OR Gibraltar* OR Greece OR Greek* OR Grenada* OR Guam OR Guamanian* OR Guatemala OR Guatemalan* OR Guinea* OR Guyan* OR Guian* OR Haiti* OR Hispaniola OR Hondura* OR Hungar* OR Indian OR Indians OR India OR Indonesia* OR Timor* OR Iran* OR Iraq* OR Isle Of Man OR Manx OR Jamaica* OR Jordan* OR Kazakh* OR Kenya* OR Korea* OR Kosovo* OR Kosovar* OR Kyrgyz* OR Kirghiz* OR Lao OR Laos OR Laotian* OR Latvia* OR Lebanon OR Lebanese OR Lesoth* OR Basutoland OR Mosotho OR Basotho OR Liberia* OR Libya* OR Lithuania* OR Macau OR Macao OR Macanese OR Macedonia* OR Madagasca* OR Malagasy Republic OR Malawi* OR Nyasaland OR Malay* OR Maldiv* OR Indian Ocean OR Mali OR Malian* OR Malta OR Maltese OR Micronesia* OR Kirabati* OR Marshall Islands OR Marshallese OR Nauru* OR Northern Mariana Islands OR Palau* OR Tuvalu* OR Mauritania OR Mauritius OR Mauritanian* OR Mauritian* OR Mexic* OR Moldov* OR Mongol* OR Montenegr* OR Morocc* OR Ifni OR Mozambique OR Mozambican* OR Portuguese East Africa OR Myanma* OR Burma OR Burmese OR Namibia* OR Nepal* OR Netherlands Antille* OR Nicaragua* OR Niger* OR Mariana* OR Oman OR Omani OR Muscat OR Pakistan* OR Palestin* OR Panama* OR Papua New Guinea* OR Paraguay* OR Peru OR Peruvian* OR Philipine* OR Philippine* OR Phillipine* OR Phillippine* OR Filipino* OR Filipina* OR Poland OR Polish OR Portug* OR "Puerto Ric*" OR Romania* OR Russia* OR Ussr OR Soviet* OR Rwand* OR Ruand* OR Samoa* OR Pacific Islands OR Polynesia OR Samoan Islands OR Sao Tome And Principe OR Sao Tomean* OR Santomean* OR Saudi* OR Senegal* OR Serbia* OR Seychell* OR "Sierra Leone*" OR Slovak* OR Slovenia* OR Melanesia* OR "Solomon Island*" OR "Norfolk Island*" OR Somali* OR "South Africa*" OR "South Sudan*" OR "Sri Lanka*" OR Ceylon* OR Saint Kitts And Nevis OR St Kitts And Nevis OR Kittitian* OR Nevisian* OR "Saint Lucia*" OR St Lucia OR Saint Vincent OR St Vincent OR Vincentian* OR Grenadines OR Sudan OR Sudanese OR Surinam* OR Syria OR Syrian* OR Tajik* OR Tadjik* OR Tadzhik* OR Tanzania* OR Tanganyika* OR Thai OR Thailand OR Siam OR Timor Leste OR East Timor OR Timorese* OR Togo OR Togolese OR Tonga OR Tongan* OR Trinidad* OR Tobago* OR Tunisia* OR Turkey OR Turkmen* OR Uganda* OR Ukrain* OR Uruguay* OR Uzbek* OR Vanuatu* OR "New Hebride*" OR Venezuela* OR Vietnam* OR Viet Nam OR Middle East OR West Bank OR Gaza OR Yemen* OR Yugoslav* OR Zambia* OR Zimbabwe* OR "Northern Rhodesia*")

OR

AB(Afghan* OR Albania* OR Algeria OR "American Samoa*" OR Angola* OR Antigua* OR Barbuda* OR Argentin* OR Armenia* OR Aruba* OR Azerbaijan* OR Bahrain* OR Bangladesh* OR Bangalees OR Bajan* OR Barbados OR Belarus* OR Byelarus* OR Belorussia* OR Byelorussia* OR Belize* OR British Honduras OR Benin* OR Dahomey OR Bhutan* OR Bolivia* OR Bosnia* OR Hercegovina* OR Herzegovina* OR Botswana* OR Batswana OR Brazil* OR Brasil* OR Bulgaria OR Burkina Faso OR Burkina Fasso OR Burkinabe OR Burkinese OR Upper Volta OR Burundi* OR Urundi OR Cabo Verde* OR Cape Verde* OR Cambodia* OR Kampuchea OR Khmer OR Cameroon* OR Cameron* OR Cameroun* OR "Central African" OR Ubangi Shari OR Chad OR Chadian* OR Chile* OR China OR Chinese OR Colombia* OR Comoros OR Comores OR Comoro Islands OR Comorian* OR Mayotte OR Congo OR Congolese OR Zaire OR Costa Rica* OR Cote D’ivoire OR Cote D’ Ivoire OR Cote Divoire OR Cote D Ivoire OR Ivory Coast OR Ivorian* OR Croatia* OR Cuba OR Cuban* OR Cyprus OR Cypriot* OR Czech* OR Djibouti* OR French Somaliland OR Dominica* OR Ecuador* OR Egypt* OR United Arab Republic OR El Salvador OR Salvadoran* OR "Equatorial Guinea*" OR Equatoguinean* OR Spanish Guinea OR Eritrea* OR Estonia* OR Eswatini OR Swaziland OR Swazi OR Swazis OR Swati OR Swatis OR Ethiopia* OR Fiji* OR Gabon* OR Gambia* OR Georgia* OR Ghana* OR Gold Coast OR Gibraltar* OR Greece OR Greek* OR Grenada* OR Guam OR Guamanian* OR Guatemala OR Guatemalan* OR Guinea* OR Guyan* OR Guian* OR Haiti* OR Hispaniola OR Hondura* OR Hungar* OR Indian OR Indians OR India OR Indonesia* OR Timor* OR Iran* OR Iraq* OR Isle Of Man OR Manx OR Jamaica* OR Jordan* OR Kazakh* OR Kenya* OR Korea* OR Kosovo* OR Kosovar* OR Kyrgyz* OR Kirghiz* OR Lao OR Laos OR Laotian* OR Latvia* OR Lebanon OR Lebanese OR Lesoth* OR Basutoland OR Mosotho OR Basotho OR Liberia* OR Libya* OR Lithuania* OR Macau OR Macao OR Macanese OR Macedonia* OR Madagasca* OR Malagasy Republic OR Malawi* OR Nyasaland OR Malay* OR Maldiv* OR Indian Ocean OR Mali OR Malian* OR Malta OR Maltese OR Micronesia* OR Kirabati* OR Marshall Islands OR Marshallese OR Nauru* OR Northern Mariana Islands OR Palau* OR Tuvalu* OR Mauritania OR Mauritius OR Mauritanian* OR Mauritian* OR Mexic* OR Moldov* OR Mongol* OR Montenegr* OR Morocc* OR Ifni OR Mozambique OR Mozambican* OR Portuguese East Africa OR Myanma* OR Burma OR Burmese OR Namibia* OR Nepal* OR Netherlands Antille* OR Nicaragua* OR Niger* OR Mariana* OR Oman OR Omani OR Muscat OR Pakistan* OR Palestin* OR Panama* OR Papua New Guinea* OR Paraguay* OR Peru OR Peruvian* OR Philipine* OR Philippine* OR Phillipine* OR Phillippine* OR Filipino* OR Filipina* OR Poland OR Polish OR Portug* OR "Puerto Ric*" OR Romania* OR Russia* OR Ussr OR Soviet* OR Rwand* OR Ruand* OR Samoa* OR Pacific Islands OR Polynesia OR Samoan Islands OR Sao Tome And Principe OR Sao Tomean* OR Santomean* OR Saudi* OR Senegal* OR Serbia* OR Seychell* OR "Sierra Leone*" OR Slovak* OR Slovenia* OR Melanesia* OR "Solomon Island*" OR "Norfolk Island*" OR Somali* OR "South Africa*" OR "South Sudan*" OR "Sri Lanka*" OR Ceylon* OR Saint Kitts And Nevis OR St Kitts And Nevis OR Kittitian* OR Nevisian* OR "Saint Lucia*" OR St Lucia OR Saint Vincent OR St Vincent OR Vincentian* OR Grenadines OR Sudan OR Sudanese OR Surinam* OR Syria OR Syrian* OR Tajik* OR Tadjik* OR Tadzhik* OR Tanzania* OR Tanganyika* OR Thai OR Thailand OR Siam OR Timor Leste OR East Timor OR Timorese* OR Togo OR Togolese OR Tonga OR Tongan* OR Trinidad* OR Tobago* OR Tunisia* OR Turkey OR Turkmen* OR Uganda* OR Ukrain* OR Uruguay* OR Uzbek* OR Vanuatu* OR "New Hebride*" OR Venezuela* OR Vietnam* OR Viet Nam OR Middle East OR West Bank OR Gaza OR Yemen* OR Yugoslav* OR Zambia* OR Zimbabwe* OR "Northern Rhodesia*")

OR

KW(Afghan* OR Albania* OR Algeria OR "American Samoa*" OR Angola* OR Antigua* OR Barbuda* OR Argentin* OR Armenia* OR Aruba* OR Azerbaijan* OR Bahrain* OR Bangladesh* OR Bangalees OR Bajan* OR Barbados OR Belarus* OR Byelarus* OR Belorussia* OR Byelorussia* OR Belize* OR British Honduras OR Benin* OR Dahomey OR Bhutan* OR Bolivia* OR Bosnia* OR Hercegovina* OR Herzegovina* OR Botswana* OR Batswana OR Brazil* OR Brasil* OR Bulgaria OR Burkina Faso OR Burkina Fasso OR Burkinabe OR Burkinese OR Upper Volta OR Burundi* OR Urundi OR Cabo Verde* OR Cape Verde* OR Cambodia* OR Kampuchea OR Khmer OR Cameroon* OR Cameron* OR Cameroun* OR "Central African" OR Ubangi Shari OR Chad OR Chadian* OR Chile* OR China OR Chinese OR Colombia* OR Comoros OR Comores OR Comoro Islands OR Comorian* OR Mayotte OR Congo OR Congolese OR Zaire OR Costa Rica* OR Cote D’ivoire OR Cote D’ Ivoire OR Cote Divoire OR Cote D Ivoire OR Ivory Coast OR Ivorian* OR Croatia* OR Cuba OR Cuban* OR Cyprus OR Cypriot* OR Czech* OR Djibouti* OR French Somaliland OR Dominica* OR Ecuador* OR Egypt* OR United Arab Republic OR El Salvador OR Salvadoran* OR "Equatorial Guinea*" OR Equatoguinean* OR Spanish Guinea OR Eritrea* OR Estonia* OR Eswatini OR Swaziland OR Swazi OR Swazis OR Swati OR Swatis OR Ethiopia* OR Fiji* OR Gabon* OR Gambia* OR Georgia* OR Ghana* OR Gold Coast OR Gibraltar* OR Greece OR Greek* OR Grenada* OR Guam OR Guamanian* OR Guatemala OR Guatemalan* OR Guinea* OR Guyan* OR Guian* OR Haiti* OR Hispaniola OR Hondura* OR Hungar* OR Indian OR Indians OR India OR Indonesia* OR Timor* OR Iran* OR Iraq* OR Isle Of Man OR Manx OR Jamaica* OR Jordan* OR Kazakh* OR Kenya* OR Korea* OR Kosovo* OR Kosovar* OR Kyrgyz* OR Kirghiz* OR Lao OR Laos OR Laotian* OR Latvia* OR Lebanon OR Lebanese OR Lesoth* OR Basutoland OR Mosotho OR Basotho OR Liberia* OR Libya* OR Lithuania* OR Macau OR Macao OR Macanese OR Macedonia* OR Madagasca* OR Malagasy Republic OR Malawi* OR Nyasaland OR Malay* OR Maldiv* OR Indian Ocean OR Mali OR Malian* OR Malta OR Maltese OR Micronesia* OR Kirabati* OR Marshall Islands OR Marshallese OR Nauru* OR Northern Mariana Islands OR Palau* OR Tuvalu* OR Mauritania OR Mauritius OR Mauritanian* OR Mauritian* OR Mexic* OR Moldov* OR Mongol* OR Montenegr* OR Morocc* OR Ifni OR Mozambique OR Mozambican* OR Portuguese East Africa OR Myanma* OR Burma OR Burmese OR Namibia* OR Nepal* OR Netherlands Antille* OR Nicaragua* OR Niger* OR Mariana* OR Oman OR Omani OR Muscat OR Pakistan* OR Palestin* OR Panama* OR Papua New Guinea* OR Paraguay* OR Peru OR Peruvian* OR Philipine* OR Philippine* OR Phillipine* OR Phillippine* OR Filipino* OR Filipina* OR Poland OR Polish OR Portug* OR "Puerto Ric*" OR Romania* OR Russia* OR Ussr OR Soviet* OR Rwand* OR Ruand* OR Samoa* OR Pacific Islands OR Polynesia OR Samoan Islands OR Sao Tome And Principe OR Sao Tomean* OR Santomean* OR Saudi* OR Senegal* OR Serbia* OR Seychell* OR "Sierra Leone*" OR Slovak* OR Slovenia* OR Melanesia* OR "Solomon Island*" OR "Norfolk Island*" OR Somali* OR "South Africa*" OR "South Sudan*" OR "Sri Lanka*" OR Ceylon* OR Saint Kitts And Nevis OR St Kitts And Nevis OR Kittitian* OR Nevisian* OR "Saint Lucia*" OR St Lucia OR Saint Vincent OR St Vincent OR Vincentian* OR Grenadines OR Sudan OR Sudanese OR Surinam* OR Syria OR Syrian* OR Tajik* OR Tadjik* OR Tadzhik* OR Tanzania* OR Tanganyika* OR Thai OR Thailand OR Siam OR Timor Leste OR East Timor OR Timorese* OR Togo OR Togolese OR Tonga OR Tongan* OR Trinidad* OR Tobago* OR Tunisia* OR Turkey OR Turkmen* OR Uganda* OR Ukrain* OR Uruguay* OR Uzbek* OR Vanuatu* OR "New Hebride*" OR Venezuela* OR Vietnam* OR Viet Nam OR Middle East OR West Bank OR Gaza OR Yemen* OR Yugoslav* OR Zambia* OR Zimbabwe* OR "Northern Rhodesia*") )

*From Advanced Search screen, make sure the boxes are* ***not*** *checked for"Apply related words", "Also search within the full text of the articles", and "Apply equivalent subjects".*

457 results as of 5/23/22

**Global Index Medicus**

|  | **Concept: Collaborative Care** |
| --- | --- |
| Subject Descriptors (MH:()) | N04.590.374.142* |
| Title, abstract, subject  (tw:()) | "integrated care"  "shared care"  "collaborative care"  "collaborative management"  ((Integrat* OR coordinat* OR collaborat* OR co-locat* OR comanage* OR co-manage*) AND care AND (health OR healthcare) AND (services OR delivery OR manag* OR systems OR model* OR organisational OR organizational OR quality))  (("disease management" OR "case management") AND (care OR health OR healthcare) AND (services OR delivery OR model* OR quality)) |

|  | **Concept: Mental Conditions** |
| --- | --- |
| Subject Descriptors | F03*  F03.600*  F03.080*  F03.084*  F03.300*  F03.700*  F03.950*  F03.900*  F01.145.805.250.250  D02.522.675  F01.700.750.300  F01.700.548  F01.145.126.980.875.600  F01.145.126.980.875.149  C10.228.140.490* |
| Title, abstract, subject  (tw:()) | depress*  anxiet*  "serious mental disorder"  "serious mental disorders"  "serious mental illness"  "serious mental illnesses"  "serious mental condition"  "serious mental conditions"  "serious mental disease"  "serious mental diseases"  "severe mental disorder"  "severe mental disorders"  "severe mental illness"  "severe mental illnesses"  "severe mental condition"  "severe mental conditions"  "severe mental disease"  "severe mental diseases"  "major mental disorder"  "major mental disorders"  "major mental illness"  "major mental illnesses"  "major mental condition"  "major mental conditions"  "major mental disease"  "major mental diseases"  "drug abuse"  "drug dependent"  "drug dependency"  "substance abuse"  "substance-related disorder"  "substance-related disorders"  "substance use disorder"  "substance use disorders"  "opioid use disorder"  "opioid use disorders"  oud  ((cocaine OR heroin OR morphine*) AND (abuse OR depend* OR withdrawal))  methadone  addict*  psychoactive*  "drug withdrawal"  "withdrawal syndrome"  "alcoholic patient"  "alcoholic patients"  "alcoholic subject"  "alcoholic subjects"  alcoholism  "alcohol dependent"  "alcohol dependency"  "alcohol-related disorder"  "alcohol-related disorders"  "chronic ethanol"  "chronic alcohol"  "alcohol withdrawal"  "ethanol withdrawal"  schizophren*  schizotyp*  schizoaffective  ((delusional OR paranoid) AND disorder*)  hallucination*  psychotic  schizoaffective  psychosis  psychoses  ((manic OR bipolar OR mood) AND disorder*)  mania*  hypomania*  "suicide attempt*"  suicidal*  "post-traumatic stress"  "posttraumatic stress"  "stress disorder"  "stress disorders"  epileps*  "seizure disorder"  "seizure disorders"  aura  auras |

|  | **Concept: Intervention Study** |
| --- | --- |
| Subject Descriptors | V03.175.250*  V03.250  V03.400  E05.318.370.150  E05.318.372.250.250* |
| Title, abstract, subject  (tw:()) | random*  controll*  "intervention study"  "experimental study"  "comparative study"  trial  trials  evaluat*  repeat*  compar*  versus  "before and after"  "interrupted time series" |

(MH:(N04.590.374.142*) OR tw:("integrated care" OR "shared care" OR "collaborative care" OR "collaborative management" OR ((Integrat* OR coordinat* OR collaborat* OR co-locat* OR comanage* OR co-manage*) AND care AND (health OR healthcare) AND (services OR delivery OR manag* OR systems OR model* OR organisational OR organizational OR quality)) OR (("disease management" OR "case management") AND (care OR health OR healthcare) AND (services OR delivery OR model* OR quality))))

**AND**

(MH:( F03* OR F03.600* OR F03.080* OR F03.084* OR F03.300* OR F03.700* OR F03.950* OR F03.900* OR F01.145.805.250.250 OR D02.522.675 OR F01.700.750.300 OR F01.700.548 OR F01.145.126.980.875.600 OR F01.145.126.980.875.149 OR C10.228.140.490*) OR TW:(depress* OR anxiet* OR "serious mental disorder" OR "serious mental disorders" OR "serious mental illness" OR "serious mental illnesses" OR "serious mental condition" OR "serious mental conditions" OR "serious mental disease" OR "serious mental diseases" OR "severe mental disorder" OR "severe mental disorders" OR "severe mental illness" OR "severe mental illnesses" OR "severe mental condition" OR "severe mental conditions" OR "severe mental disease" OR "severe mental diseases" OR "major mental disorder" OR "major mental disorders" OR "major mental illness" OR "major mental illnesses" OR "major mental condition" OR "major mental conditions" OR "major mental disease" OR "major mental diseases" OR "drug abuse" OR "drug dependent" OR "drug dependency" OR "substance abuse" OR "substance-related disorder" OR "substance-related disorders" OR "substance use disorder" OR "substance use disorders" OR "opioid use disorder" OR "opioid use disorders" OR oud OR ((cocaine OR heroin OR morphine*) AND (abuse OR depend* OR withdrawal)) OR methadone OR addict* OR psychoactive* OR "drug withdrawal" OR "withdrawal syndrome" OR "alcoholic patient" OR "alcoholic patients" OR "alcoholic subject" OR "alcoholic subjects" OR alcoholism OR "alcohol dependent" OR "alcohol dependency" OR "alcohol-related disorder" OR "alcohol-related disorders" OR "chronic ethanol" OR "chronic alcohol" OR "alcohol withdrawal" OR "ethanol withdrawal" OR schizophren* OR schizotyp* OR schizoaffective OR ((delusional OR paranoid) AND disorder*) OR hallucination* OR psychotic OR schizoaffective OR psychosis OR psychoses OR ((manic OR bipolar OR mood) AND disorder*) OR mania* OR hypomania* OR "suicide attempt*" OR suicidal* OR "post-traumatic stress" OR "posttraumatic stress" OR "stress disorder" OR "stress disorders" OR epileps* OR "seizure disorder" OR "seizure disorders" OR aura OR auras))

**AND**

(MH:(V03.175.250* OR V03.250 OR V03.400 OR E05.318.370.150 OR E05.318.372.250.250*) OR tw:( random* OR controll* OR "intervention study" OR "experimental study" OR "comparative study" OR trial OR trials OR evaluat* OR repeat* OR compar* OR versus OR "before and after" OR "interrupted time series"))

815 results as of 5/23/22

(324 in English)
